# Methods of Monitoring Internal and External Loads and Their Relationships with Physical Qualities, Injury, or Illness in Adolescent Athletes: A Systematic Review and Best-Evidence Synthesis

**DOI:** 10.1007/s40279-023-01844-x

**Published:** 2023-04-18

**Authors:** Charles Dudley, Rich Johnston, Ben Jones, Kevin Till, Harrison Westbrook, Jonathon Weakley

**Affiliations:** 1 School of Behavioural and Health Sciences, Australian Catholic University, Banyo Campus, Brisbane, Australia; 2 St Joseph’s Nudgee College, Boondall, Brisbane, Australia; 3 Carnegie Applied Rugby Research (CARR) Centre, Carnegie School of Sport, Leeds Beckett University, Leeds, UK; 4 Health through Physical Activity, Lifestyle and Sport Research Centre (HPALS), Department of Human Biology, Faculty of Health Sciences, University of Cape Town, Cape Town, South Africa; 5 Premiership Rugby, London, UK; 6 Leeds Rhinos Rugby League Club, Leeds, UK; 7 England Performance Unit, The Rugby Football League, Leeds, UK; 8 Sports Performance, Recovery, Injury and New Technologies (SPRINT) Research Centre, Australian Catholic University, Brisbane, Australia

## Abstract

**Background:**

With the increasing professionalisation of youth sports, training load monitoring is increasingly common in adolescent athletes. However, the research examining the relationship between training load and changes in physical qualities, injury, or illness in adolescent athletes is yet to be synthesised in a systematic review.

**Objective:**

The aim of this review was to systematically examine the research assessing internal and external methods of monitoring training load and physical qualities, injury, or illness in adolescent athletes.

**Methods:**

Systematic searches of SPORTDiscus, Web of Science, CINAHL and SCOPUS were undertaken from the earliest possible records to March 2022. Search terms included synonyms relevant to adolescents, athletes, physical qualities, injury, or illness. To be eligible for inclusion, articles were required to (1) be original research articles; (2) be published in a peer-reviewed journal; (3) include participants aged between 10 and 19 years and participating in competitive sport; (4) report a statistical relationship between a measure of internal and/or external load and physical qualities, injury or illness. Articles were screened and assessed for methodological quality. A best-evidence synthesis was conducted to identify trends in the relationships reported.

**Results:**

The electronic search yielded 4125 articles. Following screening and a review of references, 59 articles were included. The most commonly reported load monitoring tools were session ratings of perceived exertion (*n* = 29) and training duration (*n* = 22). Results of the best-evidence synthesis identified moderate evidence of positive relationships between resistance training volume load and improvement in strength, and between throw count and injury. However, evidence for other relationships between training load and change in physical qualities, injury, or illness were limited or inconsistent.

**Conclusions:**

Practitioners should consider monitoring resistance training volume load for strength training. Additionally, where appropriate, monitoring throw counts may be useful in identifying injury risk. However, given the lack of clear relationships between singular measures of training load with physical qualities, injury, or illness, researchers should consider multivariate methods of analysing training load, as well as factors that may mediate the load–response relationship, such as maturation.

**Supplementary Information:**

The online version contains supplementary material available at 10.1007/s40279-023-01844-x.

## Key Points

**Table:** 

The most commonly reported methods of monitoring internal load in adolescent athletes are session rating of perceived exertion (sRPE) and heart rate, whilst the most commonly reported methods of monitoring external load are training duration and global navigation satellite systems (GNSS).
There is moderate evidence of a relationship between resistance training volume and increases in strength.
There is moderate evidence of a relationship between training duration and throw count, and injuries.
All other relationships between internal and external loads and changes in physical qualities, injuries, or illness were limited or inconsistent.
It is strongly recommended that future research investigating the training load of adolescent athletes measures and reports the maturity status of the participants.

## Introduction

Training and physical activity are integral for physical development [1]. When an athlete completes a training session, there is an acute increase in fatigue, which, with recovery, is then typically followed by a supercompensatory response [2]. Improving physical qualities has previously been shown to improve physical performance [3, 4], decrease injury risk [5], improve recovery [6], and influence selection [7] in team sports, and therefore forms a significant focus of the training process. However, without adequate recovery following training, the athlete may suffer decreased performance and potentially injury or illness [8, 9]. This relationship was originally referred to as the general adaptation syndrome [2], and despite this model having undergone refinement [10], the principle of providing a sequentially greater training stimulus, followed by adequate rest and recovery, remains the premise on which most modern training programmes are based. Colloquially, this balance between fitness and fatigue has been termed the ‘Goldilocks effect’ and highlights the need to understand both the positive and negative responses to training load [11].

To ensure appropriate prescription of training and rest, load monitoring programmes are often implemented, particularly in elite sport [12]. However, with the increasing professionalisation of youth sports, greater emphasis is being placed on quantifying the training loads of adolescent athletes [13–15]. There are both internal and external methods of monitoring training loads. External methods of monitoring load measure the work performed by an athlete, including resistance training volume load (sets × reps × load) and running metrics through global navigation satellite systems (GNSS) [16]. Alternatively, internal load monitoring methods capture the physiological (e.g., heart rate; HR) and psychophysiological (e.g., session rating of perceived exertion; sRPE) responses to the external load [16]. In comparison to external load, internal load is a more accurate measurement of the individualised response to training stress [17]. However, it is challenging to prescribe training based on internal load, as this is influenced by numerous factors, for example, hydration status [18]. Therefore, it is often more practical to prescribe training based on external loads. Given the limitations of internal and external load metrics, both internal and external loads will often be integrated in a load monitoring regimen.

Throughout adolescence, an athlete's response to training load will change due to factors such as maturation and training exposure [19], and therefore they are likely to have fluctuating responses to training load. For example, changes in sex hormones throughout maturation facilitate greater strength and hypertrophy adaptations [19, 20]. Given the unique environment of adolescent athletic development, multiple attempts at developing training models to optimise adolescent athletic development have been proposed, such as the long-term athlete development model [21] and the youth physical development model [22]. These models propose that the development of certain physical qualities should be emphasised at different points throughout maturation. This highlights the need for a systematic review of the literature to understand current evidence about the complex nature of the load–response relationship in adolescent athletes.

Given the increased focus on training load monitoring in adolescent athletes, a systematic review of the literature is appropriate to guide practitioners and researchers on the relationship between methods of monitoring training load and physical qualities, injury, or illness. Subsequently, the aim of this systematic review was to detail the methods of reporting internal and external loads in adolescent athletes and describe their relationship with changes in physical qualities, injury, or illness.

## Methods

### Design and Search Strategy

This review was registered via PROSPERO (CRD42021245503). An electronic search was conducted of the CINAHL, SPORTDiscus, Web of Science, and SCOPUS databases. Search terms and strategy are reported in Table 1. Search terms were crafted by reviewing known original research and reviews relevant to the topic [23]. No searches were mapped to medical subject heading terms. The search strings were initially searched independently and then combined with AND. Strings were adjusted based on database-specific truncation, wildcard, and proximity operators. The search was restricted to studies published in English. Articles were retrieved from the earliest possible date until March 2022.

**Table 1:** Search terms used

Variable	Search strings
Adolescent	Adolescen* OR teen* OR Pubescent OR junior OR “School athlet*” OR youth* OR “Under#11” OR “Under#12” OR “Under#13” OR “Under#14” OR “Under#15” OR “Under#16” OR “Under#17” “Under#18” OR “Under#19”
Athletes	archer* OR athlete* OR baseballer* OR basketballer* OR batsm?n OR boarder* OR bobsledder* OR bowler* OR boxer* OR canoeist* OR cricketer* OR cyclist* OR dancer* OR footballer* OR golfer* OR gymnast* OR handballer* OR hurdler* OR jockey* OR kayaker* OR marathoner* OR netballer* OR orienteer* OR racewalker* OR rower* OR Rugby OR sailor* OR skater* OR skier* OR softballer* OR sportsm?n OR sportspeople OR sportsperson* OR sportswom?n OR sprinter* OR swimmer* OR volleyballer* OR weightlifter* OR wrestler* OR “badminton player*” OR “baseball player*” OR “basketball player*” OR “football player*” OR “handball player*” OR “hockey player*” OR “lacrosse player*” OR “martial artist*” OR “netball player*” OR “race walker*” OR “soccer player*” OR “softball player*” OR “squash player*” OR “tennis player*” OR “volleyball player*” OR “water polo player*” OR “weight lifter*” OR *rider* OR *runner*
Load monitoring	"Training load*" OR "Physical load*" OR "work load*" OR load* OR "Training practice*" OR "Global workload index" OR "NASA-TLX" OR "*RPE" OR "Perceived Exertion" OR trimp OR GPS OR "Training volume" OR "Training frequency"
Physical qualities	perform* OR fitness OR strength OR power OR cognitive OR aerobic OR skills OR physiolog* OR Jump OR physical N5 (Measure* OR assess* OR test* OR utility OR instrument* OR checklist* OR questionnaire* OR capacity OR perform* OR qualities)
Injuries and illness	injur* OR Illness OR “Upper respiratory tract infection” OR URTI
NOT	“systematic review” OR “Rat”

### Inclusion and Exclusion Criteria

The Preferred Reporting Items for Systematic reviews and Meta-Analyses (PRISMA) guidelines were followed to screen articles [24]. Article screening was performed by CD and JW; a third reviewer (RJ) was used to resolve any conflicts. Inclusion criteria were original research investigations, full-text articles written in English, published in a peer-reviewed academic journal, with participants aged 10–19 years old who participated in competitive sport [25]. Competitive sport was defined as any game or activity that involves physical exertion and skill, played against other teams or individuals [26]. Additionally, all studies were required to report a statistical relationship between a measure of internal or external training load and physical quality, injury or illness. Manuscripts were excluded if they were commentaries, letters, editorials, conference proceedings, case reports, conference abstracts or non–peer-reviewed articles and studies with < 1 week of load monitoring or alterations to load such as ‘shock periods’ [27].

Both observational and intervention-based studies were included, provided there was an indication of the relationship between load and change in physical quality, injury, or illness. Load was defined as “the cumulative amount of stress placed on an individual from multiple training sessions (structured or unstructured) over a period of time.” [28]. Physical quality was defined as any test of an element of fitness, such as strength, power, endurance, or speed. Illness was defined as any non-musculoskeletal medical reporting event. Additionally, injury was defined as a medical reporting event, whether or not it resulted in time loss [29]. Due to various methods of reporting injury and illness data, the definitions were deliberately kept broad. Finally, studies were included if they reported either the incidence or burden of injury (hours or sessions of training lost).

### Assessment of Study Quality

A modified Downs and Black [30] checklist was used to assess methodological quality by a single reviewer (CD) (Supplementary Material 1, see electronic supplementary material [ESM]); if clarification was required for any of the studies, a second reviewer was consulted (JW). This checklist has previously been used in sport science systematic reviews that similarly included a variety of study designs [31]. Items were scored as 1 (yes) or 0 (no or unable to determine), with a maximum score of 12.

### Data Extraction and Analysis

Data were extracted by CD from included studies into a custom Google spreadsheet (Alphabet, Mountain View, CA, USA). Extracted data included participant characteristics such as age, stature, body mass, maturation level (if reported), sport, and playing level. The study results extracted were the method of monitoring the training load, and the measurement of change in physical quality, injury, or illness. Statistical interpretations of the results were only provided if reported in the original research. Contributing findings included in the best-evidence synthesis were any reported statistical relationship from included studies. Unclear or erroneous data, such as data with multiple decimal places or implausible values, were reported, but not included in the best-evidence synthesis. Assessments of physical qualities were grouped into relevant categories, being strength, power, aerobic fitness, repeated sprint ability, flexibility, muscular endurance, and change of direction. Studies included in this systematic review included a number of different study types (i.e., intervention and observational) and different statistical methods (i.e., correlation, hypothesis testing, effect sizes). As such, the heterogeneity of the results precluded meta-analysis, and data were therefore synthesised according to the following criteria [23, 32]:

*Strong evidence:* Consistent findings across two or more studies, and at least 75% of all contributing findings.

*Moderate evidence:* Consistent findings across two or more studies, and at least 50% of all contributing findings.

*Limited evidence:* Consistent findings identified in one study, and at least 50% of all contributing findings.

*Inconsistent evidence:* Conflicting findings across multiple studies, or less than 50% of contributing findings.

*No evidence:* No changes reported.

## Results

### Search Findings and Study Selection

**Fig. 1:**
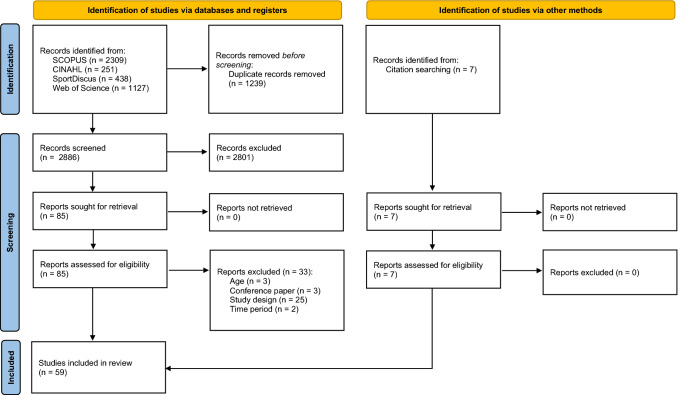
Preferred Reporting Items for Systematic reviews and Meta-Analyses (PRISMA) flow diagram of search strategy

The search results are highlighted in Fig. 1. A total of 85 full-text articles were screened, with 59 studies included in the final review.

### Research Reporting Quality

Methodological scores ranged from 6 to 11 with a mean of 8.4 ± 1.4 out of 12 (Supplementary Material 2, see ESM). No articles were excluded on the basis of methodological quality.

### Study Characteristics

Participant characteristics are presented in Table 2. Sports included cricket (*n* = 1), soccer (*n* = 19), multi-sports (*n* = 6), water polo (*n* = 1), basketball (*n* = 2), Irish dancing (*n* = 1), rugby league (*n* = 1), tennis (*n* = 7), weightlifting (*n* = 2), track & field (*n* = 4), baseball (*n* = 3), Australian football (*n* = 1), Gaelic football (*n* = 1), table tennis (*n* = 1), gymnastics (*n* = 3), rugby union (*n* = 3), volleyball (*n* = 1), and orienteering (*n* = 2). Year of publication ranged from 2002 to 2022, with 88% of studies published since 2012. Sample sizes ranged from eight to 2011 athletes (total = 8935; median = 35). In total, 35 studies investigated males, five investigated females, 18 investigated both males and females, and one did not state sex. The reported mean age of the participants ranged from 13.4 to 18.8 years. Twenty-four studies assessed internal load response, 27 assessed external loads, and eight assessed both internal and external loads. The most commonly reported internal load monitoring tools were sRPE (*n* = 29) and heart rate (*n* = 7). The most commonly reported external load monitoring tools were training duration (*n* = 22) and GNSS (*n* = 5). Physical qualities investigated included strength (*n* = 5), aerobic fitness (*n* = 19), speed (*n* = 12), power (*n* = 3), change of direction (*n* = 7), flexibility (*n* = 1), muscular endurance (*n* = 1) and repeated sprint ability (*n* = 3). Additionally, 34 studies investigated injury, and six studies investigated illness.

**Table 2:** Study and participant characteristics

References	Year	Sport and level	Sample size	Age	Sex	Stature (cm)	Weight (kg)	Monitoring tool	Outcome of interest
Akubat et al. [34]	2012	Professional soccer	9	17.0 ± 1	Male	181.0 ± 5.0	72.9 ± 6.7	sRPE Heart rate	Physical quality
Brink et al. [39]	2010	Professional soccer	18	17.0 ± 0.5	Male	180.0 ± 7.3	72.4 ± 7.8	sRPE	Physical quality
Brisola et al. [41]	2020	National water polo	20	15.7 ± 1.3	Female	162.0 ± 10.0	60.9 ± 11.0	sRPE	Physical quality Illness
Chaabene and Negra [44]	2017	Academy soccer	25	12.7 ± 0.2 (LPT) 12.7 ± 0.3 (HPT) 14.3 ± 0.3 (LPT APHV) 14.3 ± 0.8 (HPT APHV)	Male	157.2 ± 3.6 (LPT) 155.9 ± 9.0 (HPT)	42.7 ± 4.7 (LPT) 45.0 ± 8.5 (HPT)	Plyometric volume	Physical quality
Dobbin et al. [46]	2018	Academy rugby league	16	17.2 ± 0.7	Male	179.9 ± 4.9	88.5 ± 10.1	sRPE	Physical quality
Ellis et al. [92]	2020	Academy soccer	9	17.1 ± 1	Male	179.0 ± 5.6	71.3 ± 5.8	sRPE Heart rate GNSS	Physical quality
Figueiredo et al. [50]	2019	Professional soccer	16	18.7 ± 0.6	Male	175.0 ± 5.6	69.1 ± 6.6	sRPE	Physical quality
Figueireido et al. [49]	2019	Youth soccer	16	18.8 ± 0.7	Male	175.3 ± 5.5	68.7 ± 6.5	sRPE Heart rate	Physical quality
Fitzpatrick et al. [51]	2018	Professional soccer	14	17.1 ± 0.5	Male	178.3 ± 4.6	70.9 ± 5.8	sRPE GNSS	Physical quality
Gil-Rey et al. [54]	2015	Professional soccer	28	17.6 ± 0.6 (elite) 17.5 ± 0.5 (non-elite)	Male	179.7 ± 5.6 (elite) 178.1 ± 5.6 (non-elite)	70.3 ± 4.4 71.1 ± 6.5	dRPE	Physical quality
González-Badillo et al. [55]	2005	National weightlifting	51	16.4 ± 1.3 (low volume) 16.5 ± 1.4 (medium volume) 16.8 ± 1.7 (high volume)	Male	167.3 ± 3.9 (low volume) 166.7 ± 4.1 (medium volume) 165.4 ± 5.6 (high volume)	72.7 ± 5.4 (low volume) 70.5 ± 5.7 (medium volume) 69.4 ± 5.3 (high volume)	Training volume	Physical quality
González-Badillo et al. [56]	2006	National weightlifting	29	17.1 ± 1.7 (low intensity) 16.9 ± 1.7 (medium intensity) 17.5 ± 1.9 (high intensity)	Male	168.0. ± 4.1 (low intensity) 167.0 ± 4.0 (medium intensity) 169.1 ± 3.6 (high intensity)	73.7 ± 5.5 (low intensity) 74.0 ± 3.9 (medium intensity) 72.0 ± 2.3 (high intensity)	Training volume	Physical quality
Johansson et al. [60]	2022	Tennis	301	14.5 ± 2.0	Both	169.8 ± 11.2	58.3 ± 12.7	Training volume	Injury
Johansson et al. [59]	2022	Tennis	271	14.6 ± 2.0	Both	169.9 ± 10.9	58.5 ± 12.5	Training volume	Injury
Jones et al. [61]	2021	Middle-distance running	10	16.2 ± 2	Male	173.0 ± 9	55.7 ± 10.1	Training volume Heart rate	Physical quality
Mehta et al. [67]	2022	High-school baseball	49	17.9 ± 0.4	Male	181.8 ± 6.8	80.6 ± 9.1	Throw count	Injury
Lyman et al. [65]	2002	Baseball	476	12.0	Male	152.0	48.0	Throw count	Injury
Fleisig et al. [52]	2011	Baseball	481	12.0 ± 1.7	Male			Throw count	Injury
Lopez Segovia et al. [64]	2014	Professional soccer	19	18.3 ± 0.6	Male	179.5 ± 6.8	74.4 ± 8.2	Heart rate	Physical quality
Murphy et al. [70]	2015	International tennis	30	17.0 ± 1.3	Both	176.7 ± 6 (male) 170.2 ± 3.8 (female)	66.9 ± 8.6 (male) 60.5 ± 5.5 (female)	sRPE	Physical quality
Murphy et al. [71]	2015	International tennis	30	17.0 ± 1.3	Both	176.7 ± 6 (male) 170.2 ± 3.8 (female)	66.9 ± 8.6 (male) 60.5 ± 5.5 (female)	sRPE	Physical quality
Nobari et al. [73]	2020	Soccer	23	15.5 ± 0.2 1.9 ± 0.3 maturity offset	Male	172.7 ± 4.2	61.3 ± 5.6	sRPE	Physical quality
Nobari et al. [74]	2021	Soccer	23	15.5 ± 0.2	Male	172.7 ± 4.2	61.3 ± 5.62	sRPE	Physical quality
Otaegi and Arcos [76]	2020	Club-level basketball	19	14.9 ± 0.6 (U15) 15.1 ± 0.7 (U16)	Female	161.0 ± 1.0 (U15) 164.0 ± 1.0 (U16)	58.2 ± 7.6 (U15) 62.8 ± 7.2 (U16)	sRPE	Physical quality
Prieto-Gonzaelez et al. [80]	2021	Multi-sport	498	16.4 ± 2.2	Both			Training volume	Injury
Patel et al. [77]	2021	Pathway gymnastics	42	13.4 ± 2.5 (male) 13.1 ± 2.0 (female)	Both	157.7 ± 13.7 (male) 158.1 ± 5.1 (female)	47.8 ± 15.1 (male) 50.1 ± 8.8 (female	sRPE	Injury
Sawczuk et al. [84]	2018	Academy multi-sport athletes	52	17.3 ± 0.6		173.0 ± 18.2	73.7 ± 12.6	sRPE	Physical quality
Taylor et al. [86]	2018	Academy rugby union	10	18.4 ± 1.0	Male	181.3 ± 5.9	85.9 ± 13.0	sRPE Heart rate GNSS	Physical quality
Weakley et al. [91]	2019	Schoolboy rugby union	35	16.9 ± 0.4	Male	178.0 ± 7	80.1 ± 10.5	sRPE Training volume	Physical quality
Ahmun et al. [33]	2019	International cricket	39	17.5 ± 0.8	Male			sRPE	Injury
Albrecht et al. [35]	2020	School level multi-sports	278	12.1 ± 1.2	Both			Training volume	Injury
Bacon and Mauger [37]	2017	Professional youth soccer	41	17.8 ± 1.1	Male	175.0 ± 4.5	72.4 ± 3.1	GNSS	Injury
Bowen et al. [38]	2017	Academy football	32	17.3 ± 0.9	Male	180.0 ± 7.3	74.1 ± 7.0	GNSS	Injury
Brink et al. [40]	2010	National soccer	53	16.5 ± 1.2 (season 06/07) 16.5 ± 1.1 (season 07/08)	Male	177.0 ± 7.8 (season 06/07) 177.3 ± 6.9 (season 07/08)	72.4 ± 7.8	sRPE	Injury Illness
Cahalan et al. [43]	2019	Professional Irish dancing	37	13.0–17.0^a^	4 male 33 female			Training volume	Injury
Delecroix et al. [45]	2019	Academy soccer	52	16.8 ± 0.9	Male			sRPE	Injury
Fett et al. [48]	2017	National tennis	166	DC: 15.6 ± 1.1 M: RS1 14.9 ± 2.5 F: RS1 14.6 ± 2.1 M: RS2 15.2 ± 0.6	Both	DC: 180.7 ± 9.6 M: RS1 171.2 ± 13.9 F: RS1 166.1 ± 10.9 M: RS2 176.3 ± 7.7	DC: 69.8 ± 11.7 M: RS1 58.6 ± 15.4 F: RS1 54.1 ± 10.6 M: RS2 62.4 ± 8.7	Training volume	Injury
Hartwig et al. [57]	2019	School and representative rugby union	103	15.2 ± 1.5	Male	178.0 ± 7.4	83.4 ± 9.3	Training volume	Injury
Huxley et al. [58]	2014	Professional track and field	103	17.7 ± 2.4	Both			Novel subjective scale	Injury
Kiernan et al. [62]	2018	NCAA D1 distance running	9	18.7 ± 1.0	Male	178.4 ± 4.6	629.40 ± 71.40 (N)	Accelerometer	Injury
Lathlean et al. [63]	2020	Under-18 state league ARF	290	17.3 ± 0.3	Male	188.4 ± 7.1	188.4 ± 7.1	sRPE	Injury
Martínez-Silván et al. [66]	2017	Academy middle-distance running	5	15.7 ± 1.4	Male	174.2 ± 3.2	54.2 ± 4.4	Training volume	Injury
Møller et al. [68]	2017	First division U16 and U18 soccer	679	14.0–18.0	Male			Training volume	Injury
Moreno-Pérez et al. [69]	2020	Academy tennis	15	17.2 ± 1.1	Both	178.5 ± 8.7	68.1 ± 4.8	sRPE	Injury
Myers et al. [72]	2020	Academy tennis	26	15.0 ± 2.0 16.0 ± 2.0	Both	171.0 ± 3.0 (male) 167.0 ± 2.0 (female)	61 ± 3 (male) 55 ± 3 (female)	sRPE	Injury
O'Keeffe et al. [75]	2020	Club-level Gaelic football	97	13.4 ± 1.1	Male	160.0 ± 10.0	59.3 ± 12.5	sRPE	Injury
Post et al. [79]	2017	Multi-sport athletes^b^	2011	13.5 ± 1.6 (low specialisation) 13.7 ± 1.7 (moderate specialisation) 13.8 ± 1.6 (high specialisation)	Both			Training volume	Injury
Post et al. [78]	2017	High-school athletes	1544	16.1 ± 1.1	Both			Training volume	Injury
Pullinger et al. [81]	2019	National-level table tennis	8	14.5 ± 1.4	Male	166.7 ± 6.6 − 0.6 ± 1.7 (PHV)	53.6 ± 7.9	Training volume Heart rate	Injury
Purnell et al. [82]	2010	Recreational and competitive acrobatic gymnasts	73	13.4 ± 3.6 20.5 ± 4.2	Both			Training volume	Injury
Raya-González et al. [83]	2019	Professional soccer	22	18.6 ± 0.6	Male	178.0 ± 4.0	72.2 ± 6.9	sRPE	Injury
Sugimoto et al. [85]	2019	Multi-sport athletes	236	15.3 ± 1.6 (single sport) 14.3 ± 1.7 (multi-sport)	Female	164.4 ± 8.4 (single sport) 163.0 ± 7.4 (multi-sport)	59.5 ± 12.0 (single sport) 55.5 ± 10 (mult-sport)	Volume	Injury
Visnes and Bahr [87]	2013	High-school volleyball	141	16.8 ± 0.8	Both	187.0 ± 5.5 (healthy men) 186.0 ± 6.7 (injured men) 171.8 ± 6.5 (healthy women) 173.9 ± 6.7 (injured women)	75.3 ± 7.8 (healthy men) 76.3 ± 8.5 (injured men) 65.2 ± 7.5 (healthy women) 66.0 ± 13.0 (injured women)	Training volume	Injury
Von Rosen et al. [89]	2017	National orienteers	64	17.0	Both			Training volume	Injury
Von Rosen et al. [88]	2016	National orienteers	64	17.0 ± 1.0	Both			Training volume	Injury
Watson et al. [90]	2017	Soccer^b^	75	15.5 ± 1.6	Female	164.7 ± 6.6	57.3 ± 8.2	sRPE	Injury Illness
Antualpa et al. [36]	2018	State rhythmic gymnasts	23	12.1 ± 2.6	Female	143.9 ± 13.7	37.2 ± 9.4	sRPE	Illness
Brunelli et al. [42]	2012	Regional basketball	12	12.7 ± 0.6	Male	170.0 ± 10.0	57.6 ± 12.6	sRPE	Illness
Freitas et al. [53]	2014	Professional soccer	17	16.0 ± 0.5	Male	181.3 ± 5.8	75.2 ± 3.1	sRPE	Illness

*APHV* age of peak height velocity, *ARF* Australian Rules Football, *DC* Davis cup, *dRPE* differential rating of perceived exertion, *F* female, *GNSS* global national satellite systems, *HPT* high plyometric training, *LPT* low plyometric training, *M* male, *NCAA D1* National College Athletics Associations Division 1, *N* Newtons, *PHV* peak height velocity, *RS* regional squad, *sRPE* session rating of perceived exertion

^a^Range

^b^No clear indication of level of athletes

### Best-Evidence Synthesis

Table 3 presents the results of the best-evidence synthesis. There was moderate evidence of a relationship between resistance training volume load and strength. Additionally, there was moderate evidence of a relationship between throw count and training duration, and injury. Evidence for all other relationships was either limited or inconsistent.

**Table 3:** Best-evidence synthesis of relationship between monitoring tools and change in physical qualities, injury or illness

	Physical qualities								Injury	Illness
	Aerobic fitness	Strength	Speed	Power	Change of direction	Flexibility	Muscular endurance	Repeated sprint ability		
External training loads										
GNSS										
Total distance	−		↑						?	
High speed running (> 5 ms)	?								?	
Player load	−									
Acceleration/deceleration load			↑						?	
Accelerometer										
Vertical ground reaction force									↑	
Strides per session									−	
Cumulative loading			−						↑	
Training duration	?	?	?	?	↑				↑↑	↑
Resistance training volume load		↑↑	?	↑						
Throw count									↑↑	
Internal training loads										
Heart rate										
iTRIMP	?									
eTRIMP			↑						↑	
bTRIMP	?									
luTRIMP	?									
TeamTRIMP	−									
sRPE	?		?	?	?	−	−	?	?	?
dRPE	↑		↓	↑						

↑↑↑ Strong positive relationship, ↑↑ moderate positive relationship, ↑ limited positive relationship, ↓↓↓ strong negative relationship, ↓↓ moderate negative relationship, ↓ limited negative relationship, ? inconsistent significant relationships, − no significant relationship reported, *bTRIMP* Banister’s training impulse, *dRPE* differential ratings of perceived exertion, *eTRIMP* Edwards’ training impulse, *GNSS* global navigation satellite system, *iTRIMP* individualised training impulse, *luTRIMP* Lucia’s training impulse, *sRPE* session rating of perceived exertion, *TeamTRIMP* team training impulse

### External Training Loads

#### Relationship Between External Training Loads and Physical Qualities

Table 4 presents the relationships between external training loads and physical qualities. Nineteen studies investigated the relationship between external training loads and physical qualities [34, 39, 44, 47, 48, 51, 54–56, 64, 76, 91, 93, 94]; only one reported no significant relationships [44].

**Table 4:** Results of external methods of monitoring load and relationship with change in physical qualities

Monitoring method	Measure	Relationship	References
GNSS	Acceleration/deceleration load vs MAS	*r* = 0.20 [90% CI − 0.29 to 0.60]	[51]
	Acceleration/deceleration load vs maximal sprint speed	*r* = 0.57 [90% CI 0.15 to 0.81]; *R*^2^ = 0.32	[51]
	Distance > 15 km/h vs velocity at lactate threshold	*r =* − 0.06 [99% CI − 0.77 to 0.72]; *p* = 0.87	[86]
	Distance > 15 km/h vs velocity at *V̇*O_2max_	*r =* 0.32 [99% CI − 0.57 to 0.86]; *p* = 0.36	[86]
	Distance > 15 km/h vs V̇O_2max_	*r =* − 0.19 [99% CI − 0.82 to 0.65]; *p* = 0.59	[86]
	Distance > 15 km/h vs vOBLA	*r =* 0.25 [99% CI − 0.62 to 0.87]; *p* = 0.49	[86]
	Distance > 18 km/h vs velocity at *V̇*O_2max_	*r =* − 0.16 [99% CI − 0.81 to 0.67]; *p* = 0.66	[86]
	Distance > 18 km/h vs vLT	*r =* − 0.43 [99% CI − 0.89 to 0.22]; *p* = 0.22	[86]
	Distance > 18 km/h vs V̇O_2max_	*r =* − 0.63 [99% CI − 0.94 to 0.23]; *p* = 0.05	[86]
	Distance > 18 km/h vs vOBLA	*r =* − 0.66 [99% CI − 0.94 to 0.18]; *p* = 0.04*	[86]
	Distance > 21 km/h vs MAS	*r =* − 0.70 [90% CI − 0.51 to 0.40]; *R*^2^ = 0.00	[51]
	Distance > 21 km/h vs maximal sprint speed	*r =* 0.25 [90% CI − 0.24 to 0.64]; *R*^2^ = 0.06	[51]
	Distance > 25.2 km/h vs MAS	*r =* − 0.10 [95% CI − 0.74 to 0.54]; *R*^2^ = 0.12 [95% CI 0.00 to 0.39],	[92]
	Distance > 25.2 km/h vs speed at 2 mmol/L	*r =* − 0.22 [95% CI − 0.80 to 0.43]; *R*^2^ = 0.15 [95% CI 0.00 to 0.44]	[92]
	Distance > 25.2 km/h vs speed at 4 mmol/L	*r =* − 0.15 [95% CI − 0.76 to 0.49]; *R*^2^ = 0.13 [95% CI 0.00 to 0.42]	[92]
	Distance > 30% ASR vs MAS	*r =* 0.20 [90% CI − 0.28 to 0.61]; *R*^2^ = 0.04	[51]
	Distance > 30% ASR vs maximal sprint speed	*r =* − 0.09 [90% CI − 0.53 to 0.39]; *R*^2^ = 0.01	[51]
	Distance > MAS vs MAS	*r =* 0.5 [90% CI − 0.6 to 0.78]; *R*^2^ = 0.25	[51]
	Distance > MAS vs maximal sprint speed	*r =* 0.30 [90% CI − 0.18 to 0.67]; *R*^2^ = 0.25	[51]
	Distance > speed at 4 mmol/L vs MAS	*r =* 0.27 [95% CI − 0.37 to 0.82]; *R*^2^ = 0.16 [95% CI 0.00 to 0.47]	[92]
	Distance > speed at 4 mmol/L vs speed at 2 mmol/L	*r =* − 0.01 [95% CI − 0.73 to 0.56]; *R*^2^ = 0.12 [95% CI 0.00 to 0.40]	[92]
	Distance > speed at 4 mmol/L vs speed at 4 mmol/L	*r =* − 0.12 [95% CI − 0.71 to 0.56]; *R*^2^ = 0.12 [95% CI 0.00 to 0.40]	[92]
	Distance > vOBLA vs velocity at V̇O_2max_	*r =* 0.34 [99% CI − 0.55 to 0.87]; *p* = 0.33	[86]
	Distance > vOBLA vs vLT	*r =* 0.12 [99% CI − 0.70 to 0.80]; *p* = 0.75	[86]
	Distance > vOBLA vs V̇O_2max_	*r =* − 0.26 [99% CI − 0.85 to 0.61]; *p* = 0.47	[86]
	Distance > vOBLA vs vOBLA	*r =* 0.27 [99% CI − 0.61 to 0.85]; *p* = 0.46	[86]
	Distance between 14.4 and 19.8 km/h vs MAS	*r =* 0.11 [95% CI − 0.52 to 0.73]; R^2^ = 0.12 [95% CI 0.00 to 0.39]	[92]
	Distance between 14.4 and 19.8 km/h vs speed at 2 mmol/L	*r =* − 0.45 [95% CI − 0.90 to 0.17]; *R*^2^ = 0.27 [95% CI 0.00 to 0.57]	[92]
	Distance between 14.4 and 19.8 km/h vs speed at 4 mmol/L	*r =* − 0.45 [95% CI − 0.89 to 0.19]; *R*^2^ = 0.27 [95% CI 0.00 to 0.56]	[92]
	Distance between 19.8 and 25.2 km/h vs MAS	*r =* − 0.06 [95% CI − 0.69 to 0.58]; *R*^2^ = 0.12 [95% CI 0.00 to 0.39]	[92]
	Distance between 19.8 and 25.2 km/h vs speed at 2 mmol/L	*r =* − 0.25 [95% CI − 0.81 to 0.41]; *R*^2^ = 0.18 [95% CI 0.00 to 0.49]	[92]
	Distance between 19.8 and 25.2 km/h vs speed at 4 mmol/L	*r =* − 0.33 [95% CI − 0.86 to 0.32]; *R*^2^ = 0.22 [95% CI 0.00 to 0.54]	[92]
	Distance vs MAS	*r =* 0.34 [95% CI − 0.30 to 0.85]; *R*^2^ = 0.21 [95% CI 0.00 to 0.51]	[92]
	Distance vs MAS	*r =* 0.26 [90% CI − 0.23 to 0.64]	[51]
	Distance vs maximal sprint speed	*r =* 0.46 [90% CI 0.00 to 0.76]; *R*^2^ = 0.21	[51]
	Distance vs speed at 4 mmol/L	*r =* − 0.11 [95% CI 0.74 to 0.54]^a^; *R*^2^ = 0.11 [95% CI 0.00 to 0.37]	[92]
	Distance vs velocity at 2 mmol/L	*r =* − 0.14 [95% CI − 0.74 to 0.51]; R^2^ = 0.12 [95% CI 0.00 to 0.40]	[92]
	Distance vs velocity at V̇O_2max_	*r =* − 0.002 [99% CI − 0.75 to 0.75]; *p* = 0.99	[86]
	Distance vs vLT	*r =* − 0.21 [99% CI − 0.83 to 0.64]; *p* = 0.56	[86]
	Distance vs V̇O_2max_	*r =* − 0.51 [99% CI − 0.91 to 0.39]; *p* = 0.13	[86]
	Distance vs vOBLA	*r =* − 0.31 [99% CI − 0.86 to 0.57]; *p* = 0.38	[86]
	Player load vs MAS	*r =* 0.56 [95% CI − 0.34 to 0.94]; *R*^2^ = 0.38 [95% CI 0.01 to 0.63]	[92]
	Player load vs speed at 2 mmol/L	*r =* 0.49 [95% CI − 0.13 to 0.90]; R^2^ = 0.30 [95% CI 0.01 to 0.58]	[92]
	Player load vs speed at 4 mmol/L	*r =* 0.51 [95% CI − 0.10 to 0.92]; *R*^2^ = 0.31 [95% CI 0.00 to 0.59]	[92]
	Player load vs velocity at V̇O_2max_	*r =* − 0.17 [99% CI − 0.67 to 0.82]; *p* = 0.64	[86]
	Player load vs vLT	*r =* − 0.03 [99% CI − 0.76 to 0.74]; *p* = 0.93	[86]
	Player load vs V̇O_2max_	*r =* − 0.24 [99% CI − 0.84 to 0.62]; *p* = 0.5	[86]
	Player load vs vOBLA	*r =* − 0.47 [99% CI − 0.9 to 0.43]; *p* = 0.17	[86]
	Time > 17 km/h vs MAS	*r =* 0.22 [90% CI − 0.27 to 0.62]; *R*^2^ = 0.05	[51]
	Time > 17 km/h vs MAS	*r =* 0.37 [90% CI − 0.17 to 0.68]; *R*^2^ = 0.14	[51]
	Time > 17 km/h vs maximal sprint speed	*r =* 0.34 [90% CI − 0.15 to 0.69]; *R*^2^ = 0.11	[51]
	Time > 21 km/h vs MAS	*r =* 0.05 [90% CI − 0.42 to 0.50]; *R*^2^ = 0.14	[51]
	Time > 21 km/h vs maximal sprint speed	*r =* 0.27 [90% CI − 0.22 to 0.65]; *R*^2^ = 0.07	[51]
	Time > 30% ASR vs MAS	*r =* 0.62 [90% CI 0.22 to 0.84]; R^2^ = 0.38	[51]
	Time > 30% ASR vs maximal sprint speed	*r =* − 0.15 [90% CI − 0.57 to 0.33]; *R*^2^ = 0.02	[51]
	Time > MAS vs MAS	*r =* 0.77 [90% CI 0.48 to 0.91]; *R*^2^ = 0.59	[51]
	Time > MAS vs maximal sprint speed	*r =* 0.21 [90% CI − 0.28 to 0.61]; *R*^2^ = 0.04	[51]
Resistance training volume	High- or low-volume group vs snatch & clean and jerk in medium-volume group	No significant difference reported	[55]
	High-volume group vs snatch in medium-volume group	*p* = 0.09	[55]
	Lower-body exercises vs squat (kg)	*r =* 0.30; *p* > 0.05	[91]
	Lower-body volume load vs squat (kg)	*r =* 0.30; *p* > 0.05	[91]
	Lower-body volume load vs CMJ height	*r =* 0.74; *p* < 0.05*	[91]
	Lower-body volume load vs CMJ mean force	*r =* 0.49; *p* < 0.05*	[91]
	Lower-body volume load vs 20 m sprint	*r =* 0.19; *p* > 0.05	[91]
	Lower-body volume load vs 40 m sprint	*r =* 0.10; *p* > 0.05	[91]
	Medium-volume group compared with low-volume group vs snatch 1RM	*p* = 0.0015*	[55]
	Number of lifts performed at 100% 1RM in the snatch in the medium-intensity and high-intensity groups vs snatch 1RM	*r =* 0.52; *p* = 0.015*	[56]
	Number of lifts performed at 100% 1RM in the squat in the medium-intensity and high-intensity groups vs squat 1RM	*r =* 0.47; *p* = 0.03*	[56]
	Number of lifts performed at 90–100% 1RM in the clean and jerk in the medium-intensity group and high-intensity group vs clean and jerk 1RM	*r =* − 0.47; *p* = 0.055	[56]
	Number of loaded jumps vs 20 m sprint	*r =* − 0.54; *p* < 0.05*	[64]
	Number of loaded jumps vs fly 10 (10–20 m of 30 m)	*r =* − 0.56; *p* < 0.05*	[64]
	Number of repetitions of squat vs 10 m sprint	*r =* − 0.56 *p* < 0.05*	[64]
	Number of repetitions of squat vs 20 m sprint	*r =* 0.58^a^; *p* < 0.05*	[64]
	Number of repetitions of squat vs 30 m sprint	*r =* − 0.56; *p* < 0.05*	[64]
	Number of repetitions of squat vs fly 10 (10–20 of 30 m)	*r =* − 0.56; *p* < 0.05*	[64]
	Number of unloaded jumps vs 20 m sprint	*r =* − 0.53; *p* < 0.05*	[64]
	Number of unloaded jumps vs 30 m sprint	*r =* − 0.53; *p* < 0.05*	[64]
	Number of unloaded jumps vs fly 10 (10–20 of 30 m)	*r =* − 0.56; *p* < 0.05*	[64]
	Plyometric volume vs CMJ	ES = 0.00; *p* = 0.95	[44]
	Plyometric volume vs squat jump	ES = 0.00; *p* = 0.96	[44]
	Plyometric volume vs standing long jump	ES = 0.00; *p* = 0.96	[44]
	Plyometric volume vs T-Test	ES = 0.39; *p* = 0.18	[44]
	Volume load vs bench press (kg)	*r =* 0.31; *p* > 0.05	[91]
	Volume load vs chin up (kg)	*r =* 0.72; *p* < 0.01*	[91]
	Volume load vs squat (kg)	*r =* 0.25; *p* > 0.05	[91]
	Upper-body exercises vs bench press (kg)	*r =* 0.41; *p* ≤ 0.05*	[91]
	Upper-body exercises vs chin up (kg)	*r =* 0.65; *p* < 0.01*	[91]
	Upper-body volume load vs bench press (kg)	*r =* 0.45; *p* < 0.01*	[91]
	Upper-body volume load vs chin up (kg)	*r =* 0.73; *p* < 0.01*	[91]
	Upper-body volume load vs 800 m time	*r =* 0.778, *p* = 0.04*	[61]
Volume (time)	Minutes training vs time to exhaustion	*r =* 0.67 [90% CI ± 0.21]	[54]
	Minutes spent resistance training vs 20 m sprint (%)	*r =* 0.26; *p* > 0.05	[91]
	Minutes spent resistance training vs 40 m sprint (%)	*r =* 0.04; *p* > 0.05	[91]
	Minutes spent resistance training vs bench press 3RM (kg)	*r =* 0.19; *p* > 0.05	[91]
	Minutes spent resistance training vs chin up 3RM (kg)	*r =* 0.33; *p* > 0.05	[91]
	Minutes spent resistance training vs CMJ height (%)	*r =* 0.18; *p* > 0.05	[91]
	Minutes spent resistance training vs CMJ mean force (%)	*r =* 0.16; *p* > 0.05	[91]
	Minutes spent resistance training vs squat 3RM (kg)	*r =* 0.24; *p* > 0.05	[91]
	Minutes training (Under 15) vs 15 m sprint	*r =* 0.63 ± 0.45	[76]
	Minutes training (Under 15) vs 5 m sprint	*r =* 0.72 ± 0.38	[76]
	Minutes training (Under 15) vs CMJ height	*r =* − 0.70 ± 0.40	[76]
	Minutes training (Under 15) vs T-Test	*r =* 0.61 ± 0.46	[76]
	Minutes training (Under 15) vs YoYoIR1	*r =* − 0.74 ± 0.36	[76]
	Minutes training (Under 16) vs 15 m sprint	*r =* 0.54 ± 0.43	[76]
	Minutes training (Under 16) vs 5 m sprint	*r =* 0.52 ± 0.44	[76]
	Minutes training (Under 16) vs CMJ height	*r =* 0.39 ± 0.49	[76]
	Minutes training (Under 16) vs T-Test	*r =* 0.31 ± 0.51	[76]
	Minutes training (Under 16) vs YoYoIR1	*r =* − 0.03 ± 0.52	[76]
	Hours spent physical training vs grip strength	*R =* 0.64; *p* = 0.03*	[48]
	Hours training vs HR in submax shuttle run	1 h of training = − 0.9 beats/min change	[39]

^*^Statistically significant result

^a^Inconsistent or erroneous datum

*ASR* anaerobic speed reserve, *bTRIMP* Banister’s training impulse, *CMJ* countermovement jump, *eTRIMP* Edwards’ training impulse, *GNSS* global navigation satellite system, *HSR* high speed running, *IHSR* individualised high speed running, *iTRIMP* individualised training impulse, *luTRIMP* Lucia’s training impulse, *MAS* maximal aerobic speed, *RM* repetition maximum, *VHSR* very high-speed running, *vOBLA* velocity at onset of blood lactate accumulation, *vLT* velocity at lactate threshold, *Yo-Yo IR1* Yo-Yo Intermittent recovery test level 1

There was inconsistent or limited evidence of a relationship between GNSS metrics with change in physical qualities. Significant results were found for positive [51] and negative [94] relationships between high-speed running and changes in aerobic fitness, and a positive relationship for acceleration/deceleration and total distance with changes in sprint speed [51].

Training duration showed inconsistent evidence of a relationship with changes in physical qualities. Results for training duration were non-significant [54], negative [76], and positive [39] with aerobic fitness; non-significant [91] and negative [76] for power; non-significant [91] and negative [54] for speed; inconsistent for change of direction [76]; and non-significant [91] and positive [48] for strength.

Resistance training metrics showed inconsistent evidence of a relationship to changes in speed, but there was moderate evidence of relationship to changes in strength. Relationships between resistance training metrics and speed were non-significant [44, 91], or irregular [64]. Relationships with strength were positive between chin up 3 repetition maximum (RM) and upper body exercises, upper body volume (sets × reps × mass [kg]), and total (upper and lower body) volume [91], positive between bench press 3RM and upper body exercises and upper body volume [91], positive for snatch 1RM and total volume between medium and low volume groups [55], and positive for snatch and squat 1RM and number of lifts performed at 100% 1RM [56]. Relationships with power were observed to be non-significant for plyometrics volume measured via number of contacts [44], and positive for lower body exercises, lower body volume, and total volume [91]. Additionally, one study found upper-body resistance training volume to be related to 800-m time [61].

#### Relationship Between External Training Loads and Injury

The relationships between external training load and injury are shown in Table 5. There was inconsistent or limited evidence of a relationship between external training loads and injury. Twenty-two studies found significant relationships [35, 37–39, 52, 57, 59, 60, 62, 65, 66, 68, 77–82, 87–89, 94], whilst three had non-significant findings [43, 58, 85]. Of the studies that found significant results, one found that greater training load decreased the risk of injury in at least one variable [35]. The remaining 21 studies found greater training load, in at least one variable, was associated with increased injury risk [37–39, 57, 62, 66, 68, 78, 79, 81, 82, 87–89, 94]. However, when pooled, < 50% of contributing findings were significant.

**Table 5:** Results of external methods of monitoring training load and relationship with injury

Monitoring method	Measure vs injury risk	Relationship	References
Accelerometer	Mean estimated peak vGRF	*p* = 0.01*	[62]
	Mean number of strides per training session	*p* = 0.091	[62]
	Mean weighted cumulative loading per session	*p* < 0.01*	[62]
GNSS	2-week cumulative HSR distance 1 standard deviation above mean	OR = 0.580 [95% CI 0.330–1.021]; *p* = 0.059	[37]
	2-week cumulative HSR distance 1 standard deviation below mean	OR = 0.993 [95% CI 0.381–2.588]; *p* = 0.989	[37]
	2-week cumulative total distance 1 standard deviation above mean	OR = 0.670 [95% CI 0.395–1.137]; *p* = 0.137	[37]
	2-week cumulative total distance 1 standard deviation below mean	OR = 1.264 [95% CI 0.164–9.769]; *p* = 0.822	[37]
	3-week cumulative HSR distance 1 standard deviation above mean	OR = 1.049 [95% CI 0.543–2.029]; *p* = 0.886	[37]
	3-week cumulative HSR distance 1 standard deviation below mean	OR = 0.506 [95% CI 0.212–1.206]; *p* = 0.124	[37]
	3-week cumulative total distance 1 standard deviation above mean	OR = 0.953 [95% CI 0.442–2.054]; *p* = 0.903	[37]
	3-week cumulative total distance 1 standard deviation below mean	OR = 0.688 [95% CI 0.290–1.635]; *p* = 0.397	[37]
	4-week cumulative HSR distance 1 standard deviation above mean	OR = 1.049 [95% CI 0.543–2.029]; *p* = 0.886	[37]
	4-week cumulative HSR distance 1 standard deviation below mean	OR = 0.506 [95% CI 0.212–1.206]; *p* = 0.124	[37]
	4-week cumulative total distance 1 standard deviation above mean	OR = 0.953 [95% CI 0.442–2.054]; *p* = 0.903	[37]
	4-week cumulative total distance 1 standard deviation below mean	OR = 0.688 [95% CI 0.290–1.635]; *p* = 0.397	[37]
	High 1-week accelerations	RR = 1.83; *p* < 0.05*	[38]
	High 1-week distance > 20 km/h	RR = 0.59; *p* > 0.05	[38]
	High 1-week total distance	RR = 1.57; *p* > 0.05	[38]
	High 2-week accelerations	RR = 1.37; *p* > 0.05	[38]
	High 2-week distance > 20 km/h	RR = 1.45; *p* > 0.05	[38]
	High 2-week total distance	RR = 1.27; *p* > 0.05	[38]
	High 3-week accelerations	RR = 1.38; *p* > 0.05	[38]
	High 3-week distance > 20 km/h	RR = 1.66; *p* < 0.05*	[38]
	High 3-week total distance	RR = 1.31; *p* > 0.05	[38]
	High 4-week accelerations	RR = 1.66; *p* < 0.05*	[38]
	High 4-week accelerations ACWR	RR = 1.44; *p* > 0.05	[38]
	High 4-week accelerations ACWR with high chronic workload	RR = 1.1; *p* > 0.05	[38]
	High 4-week accelerations ACWR with low chronic workload	RR = 1.7; *p* > 0.05	[38]
	High 4-week distance > 20 km/h	RR = 1.26; *p* > 0.05	[38]
	High 4-week distance > 20 km/h ACWR	RR = 0.98; *p* > 0.05	[38]
	High 4-week distance > 20 km/h ACWR with high chronic workload	RR = 0.50; *p* > 0.05	[38]
	High 4-week distance > 20 km/h ACWR with low chronic workload	RR = 1.82; *p* > 0.05	[38]
	High 4-week total distance	RR = 1.64; *p* < 0.05*	[38]
	High 4-week total distance ACWR	RR = 1.13; *p* > 0.05	[38]
	High 4-week total distance ACWR with high chronic workload	RR = 1.21; *p* > 0.05	[38]
	High 4-week total distance ACWR with low chronic workload	RR = 1.76; *p* > 0.05	[38]
	HSR	R^2^ = 0.025; *p* = 0.323	[37]
	Low 1-week accelerations	RR = 0.35; *p* < 0.05	[38]
	Low 1-week distance > 20 km/h	RR = 0.38; *p* < 0.05*	[38]
	Low 1-week total distance	RR = 0.25; *p* < 0.001*	[38]
	Low 2-week accelerations	RR = 0.51; *p* > 0.05	[38]
	Low 2-week distance > 20 km/h	RR = 0.30; *p* < 0.05*	[38]
	Low 2-week total distance	RR = 0.62; *p* > 0.05	[38]
	Low 3-week accelerations	RR = 0.63; *p* > 0.05	[38]
	Low 3-week distance > 20 km/h	RR = 0.67; *p* > 0.05	[38]
	Low 3-week total distance	RR = 0.53; *p* > 0.05	[38]
	Low 4-week accelerations	RR = 0.93; *p* > 0.05	[38]
	Low 4-week accelerations ACWR	RR = 0.85; *p* > 0.05	[38]
	Low 4-week accelerations ACWR with high chronic workload	RR = 0.71; *p* > 0.05	[38]
	Low 4-week accelerations ACWR with low chronic workload	RR = 0.29; *p* < 0.05*	[38]
	Low 4-week distance > 20 km/h	RR = 0.79; *p* > 0.05	[38]
	Low 4-week distance > 20 km/h ACWR	RR = 0.47; *p* < 0.05*	[38]
	Low 4-week distance > 20 km/h ACWR with high chronic workload	RR = 1.52; *p* > 0.05	[38]
	Low 4-week distance > 20 km/h ACWR with low chronic workload	RR = 0.47; *p* > 0.05	[38]
	Low 4-week total distance	RR = 0.89; *p* > 0.05	[38]
	Low 4-week total distance ACWR	RR = 1; *p* > 0.05	[38]
	Low 4-week total distance ACWR with high chronic workload	RR = 0.91; *p* > 0.05	[38]
	Low 4-week total distance ACWR with low chronic workload	RR = 0.28; *p* < 0.05*	[38]
	Moderate-high 1-week accelerations	RR = 1; *p* > 0.05	[38]
	Moderate-high 1-week distance > 20 km/h	RR = 1.73; *p* < 0.05*	[38]
	Moderate-high 1-week total distance	RR = 0.95; *p* > 0.05	[38]
	Moderate-high 2-week accelerations	RR = 1.21; *p* > 0.05	[38]
	Moderate-high 2-week distance > 20 km/h	RR = 1.72; *p* < 0.05*	[38]
	Moderate-high 2-week total distance	RR = 1.55; *p* < 0.05*	[38]
	Moderate-high 3-week accelerations	RR = 1.32; *p* > 0.05	[38]
	Moderate-high 3-week distance > 20 km/h	RR = 1.15; *p* > 0.05	[38]
	Moderate-high 3-week total distance	RR = 1.36; *p* > 0.05	[38]
	Moderate-high 4-week accelerations	RR = 1.01; *p* > 0.05	[38]
	Moderate-high 4-week accelerations ACWR	RR = 1.15; *p* > 0.05	[38]
	Moderate-high 4-week accelerations ACWR with high chronic workload	RR = 1.25; *p* > 0.05	[38]
	Moderate-high 4-week accelerations ACWR with low chronic workload	RR = 0.94; *p* > 0.05	[38]
	Moderate-high 4-week distance > 20 km/h	RR = 1.56; *p* < 0.05*	[38]
	Moderate-high 4-week distance > 20 km/h ACWR	RR = 1.32; *p* > 0.05	[38]
	Moderate-high 4-week distance > 20 km/h ACWR with high chronic workload	RR = 1.27; *p* > 0.05	[38]
	Moderate-high 4-week distance > 20 km/h ACWR with low chronic workload	RR = 1.3; *p* > 0.05	[38]
	Moderate-high 4-week total distance	RR = 1.19; *p* > 0.05	[38]
	Moderate-high 4-week total distance ACWR	RR = 0.97; *p* > 0.05	[38]
	Moderate-high 4-week total distance ACWR with high chronic workload	RR = 1.19; *p* > 0.05	[38]
	Moderate-high 4-week total distance ACWR with low chronic workload	RR = 0.97; *p* > 0.05	[38]
	Moderate-low 1-week accelerations	RR = 1.01; *p* > 0.05	[38]
	Moderate-low 1-week distance > 20 km/h	RR = 1.16; *p* > 0.05	[38]
	Moderate-low 1-week total distance	RR = 1.38; *p* > 0.05	[38]
	Moderate-low 2-week accelerations	RR = 0.92; *p* > 0.05	[38]
	Moderate-low 2-week distance > 20 km/h	RR = 0.81; *p* > 0.05	[38]
	Moderate-low 2-week total distance	RR = 0.76; *p* > 0.05	[38]
	Moderate-low 3-week accelerations	RR = 0.77; *p* > 0.05	[38]
	Moderate-low 3-week distance > 20 km/h	RR = 0.84; *p* > 0.05	[38]
	Moderate-low 3-week total distance	RR = 1.23; *p* > 0.05	[38]
	Moderate-low 4-week accelerations	RR = 0.82; *p* > 0.05	[38]
	Moderate-low 4-week accelerations ACWR	RR = 1.16; *p* > 0.05	[38]
	Moderate-low 4-week accelerations ACWR with high chronic workload	RR = 1.04; *p* > 0.05	[38]
	Moderate-low 4-week accelerations ACWR with low chronic workload	RR = 1.49; *p* > 0.05	[38]
	Moderate-low 4-week distance > 20 km/h	RR = 0.73; *p* > 0.05	[38]
	Moderate-low 4-week distance > 20 km/h ACWR	RR = 1.10; *p* > 0.05	[38]
	Moderate-low 4-week HSR distance ACWR with high chronic workload	RR = 1.11; *p* > 0.05	[38]
	Moderate-low 4-week HSR distance ACWR with low chronic workload	RR = 0.86; *p* > 0.05	[38]
	Moderate-low 4-week total distance	RR = 0.73; *p* > 0.05	[38]
	Moderate-low 4-week total distance ACWR	RR = 1.25; *p* > 0.05	[38]
	Moderate-low 4-week total distance ACWR with high chronic workload	RR = 0.98; *p* > 0.05	[38]
	Moderate-low 4-week total distance ACWR with low chronic workload	RR = 1.43; *p* > 0.05	[38]
	Total distance	R^2^ = 0.14; *p* = 0.015	[37]
	Very high 1-week accelerations	RR = 3.06; *p* < 0.05*	[38]
	Very high 1-week distance > 20 km/h	RR = 0.82; *p* > 0.05	[38]
	Very high 1-week total distance	RR = 2.59; *p* > 0.05	[38]
	Very high 2-week accelerations	RR = 3.19; *p* < 0.05*	[38]
	Very high 2-week distance > 20 km/h	RR = 0.00; *p* > 0.05	[38]
	Very high 2-week total distance	RR = 2.88; *p* > 0.05	[38]
	Very high 3-week accelerations	RR = 3.84; *p* < 0.05*	[38]
	Very high 3-week distance > 20 km/h	RR = 0.33; *p* > 0.05	[38]
	Very high 3-week total distance	RR = 2.37; *p* > 0.05	[38]
	Very high 4-week accelerations	RR = 2.37; *p* > 0.05	[38]
	Very high 4-week accelerations ACWR	RR = 2.09; *p* > 0.05	[38]
	Very high 4-week accelerations ACWR with high chronic workload	RR = 2.71; *p* > 0.05	[38]
	Very high 4-week distance > 20 km/h	RR = 0.33; *p* > 0.05	[38]
	Very high 4-week distance > 20 km/h ACWR	RR = 0.95; *p* > 0.05	[38]
	Very high 4-week distance > 20 km/h ACWR with high chronic workload	RR = 1.63; *p* > 0.05	[38]
	Very high 4-week total distance	RR = 1.29; *p* > 0.05	[38]
	Very high 4-week total distance ACWR	RR = 2.09; *p* > 0.05	[38]
	Very high 4-week total distance ACWR with high chronic workload	RR = 1.8; *p* > 0.05	[38]
	Very high 4-week total distance ACWR with low chronic workload	RR = –	[38]
Throw Count	28-day rolling average	*p* = 0.014	[67]
	> 100 pitches per year	OR = 3.50 [95% CI 1.16–10.44]; *p* = 0.049*	[52]
	Game pitch count 25–49 vs elbow injury	OR = 1.03; *p* = 0.07	[65]
	Game pitch count 50–74 vs elbow injury	OR = 1.21; *p* = 0.07	[65]
	Game pitch count 75–99 vs elbow injury	OR = 1.35; *p* = 0.07	[65]
	Game pitch count 100 + vs elbow injury	OR = 1.44; *p* = 0.07	[65]
	Game pitch count 25–49 vs shoulder injury	OR = 1.15; *p* = 0.01*	[65]
	Game pitch count 50–74 vs shoulder injury	OR = 1.23; *p* = 0.01*	[65]
	Game pitch count 75–99 vs shoulder injury	OR = 1.52; *p* = 0.01*	[65]
	Game pitch count 100 + vs shoulder injury	OR = 1.77; *p* = 0.01*	[65]
Volume (time)	> 60% increase in training hours compared with 20% increase	HRR = 1.91 [1.00–3.70]; *p* = 0.05*	[68]
	2-week training time	OR = 0.98 [95% CI 0.95–1.01]; *p* = 0.04*	[35]
	2-week training time ACWR	OR = 0.87 [95% CI 0.58–1.30]; *p* = 0.91	[35]
	20–60% increase in training hours compared with 20% increase	HRR = 1.22 [0.62–2.40]; *p* = 0.57	[68]
	3-week training time	OR = 0.97 [95% CI 0.94–1.00]; *p* = 0.02*	[35]
	3-week training time ACWR	OR = 0.93 [95% CI 0.67–1.29]; *p* = 1	[35]
	4-week training time	OR = 0.97 [95% CI 0.93–1.00]; *p* = 0.02*	[35]
	4-week training time ACWR	OR = 0.90 [95% CI 0.66–1.23]; *p* = 0.57	[35]
	Beach volleyball training time	*p* = 0.8	[87]
	Competition time	*β* = − 0.701; *p* = 0.009*	[88]
	Competition time	OR = 1.41 [95% CI 1.14–1.74]; *p* = 0.001*	[57]
	Competition time per week	*d* = 0.47; *p* = 0.001*	[79]
	Fitness training time ACWR > 1.3 vs back injury	HRR = 1.13 [95% CI 1.05–1.22]; *p* = 0.15	[59]
	Fitness training time ACWR > 1.3 vs shoulder injury	HRR = 1.18 [95% CI 1.09–1.27]	[60]
	High competition time vs lower extremity risk	OR = 2.08 [95% CI 1.55–2.80]; *p* = 0.001*	[78]
	Hours playing sport	*p* < 0.001*	[79]
	Hours playing sports exceeding age	*p* = 0.002*	[79]
	Hours training vs lower extremity overuse injury	OR = 1.10 [95% CI 1.01–1.18]; *p* = 0.34	[85]
	Increased days of competition	HRR = 1.24 [95% CI 0.91–1.69]; *p* = 0.172	[89]
	Increased hours of training	HRR = 1.40 [95% CI 1.07–1.82]; *p* = 0.015*	[89]
	Individual running exposure	*r* = 0.83; *R*^2^ = 0.69*	[66]
	Individual running exposure vs time loss overuse injury risk	*r* = 0.61*	[66]
	Jump training	*p* = 0.04*	[87]
	Moderate competition volume vs lower extremity injury risk	OR = 1.68 [95% CI 1.31–2.16]; *p* < 0.001*	[78]
	Number of sets played	OR = 3.88 [95% CI 1.80–8.40]; *p* = 0.001*	[87]
	Other training	*p* = 0.26	[87]
	Strength training time	*p* = 0.7	[87]
	Tennis training time ACWR > 1.3 vs back injury	HRR = 1.17 [95% 1.06–1.28]; *p* = 0.08	[59]
	Tennis training time ACWR > 1.3 vs shoulder injury	HRR = 1.26 [95% 1.15–1.39]	[60]
	Total training time ACWR > 1.3 vs back injury	HRR = 1.18 [95% 1.07–1.30]; *p* = 0.04*	[59]
	Total training time ACWR > 1.3 vs shoulder injury	HRR = 1.22 [95% CI 1.12–1.34]	[60]
	Training hours per week at 11 years old	8 h; AUC = 0.91; *p* = 0.002*	[82]
	Training hours per week at 12 years old	8.5 h; AUC = 0.79; *p* = 0.037*	[82]
	Training hours per week at 13 years old	8.5 h; AUC = 0.78; *p* = 0.049*	[82]
	Training hours per week at 14 years old	9.75 h; AUC = 0.72; *p* = 0.083	[82]
	Training hours per week at 15 years old	12.75 h; AUC = 0.75; *p* = 0.067	[82]
	Training time	OR = 1.61 [95% CI 1.10–2.36]; *p* = 0.02*	[87]
	Training time	*p* = *0.539*	[58]
	Training time	OR = 1.03 [95% CI 0.78–1.33]; *p* = 0.84	[57]
	Training time	*β* = 0.184; *p* = 0.001*	[88]
	Training time	*d* = 0.02; *p* = 0.842	[79]
	Training time 1 week prior	OR = 1.02 [95% CI 0.98–1.05]; *p* = 0.33	[43]
	Training time 2 weeks prior	OR = 0.98 [95% CI 0.94–1.01]; *p* = 0.20	[43]
	Volleyball training time	OR = 1.72 [95% CI 1.18–2.53]; *p* = 0.005*	[87]
	Weekly training time	OR = 0.97 [95% CI 0.95–1.01]; *p* = 0.09	[35]
	Weekly training time	*R* = 0.277; [95% CI 0.096–0.409]; *p* = 0.001*	[80]
	Weekly training time	*d* = 0.19; *p* = 0.387	[79]
	Weekly training time	OR = 1.19 [95% CI 0.93–1.51]; *p* = 0.17	[57]
	Weekly training time vs overuse injury	OR = 1.07 [95% CI 0.98–1.18]; *p* ≥ 0.05	[40]
	Weekly training time vs traumatic injury	OR = 1.14 [95% CI 1.06–1.23]; *p* < 0.05*	[40]

^*^Statistically significant result

*ACWR* acute to chronic work to rest ratio, *AUC* area under the concentration–time curve, *HRR* hazard risk ratio, *HSR* high speed running, *OR* odds ratio, *RR* relative risk, *vGRF* vertical ground reaction force

For GNSS and injury risk, positive relationships with high and very high accelerations [38], and both positive [38] and negative [37] relationships with total distance were reported.

There was moderate evidence of a relationship between training duration and injury risk, with non-significant [43, 58, 85], negative [35], and positive relationships [39, 57, 59, 60, 66, 68, 78–80, 82, 85, 87–89, 94] reported. Furthermore, 56% of contributing findings indicated a positive relationship.

#### External Training Loads and Illness

The only study investigating the relationship between external training load and illness found the total duration of training and matches over a week was related to increased risk of illness that caused the withdrawal of an athlete from either training or competition (OR 1.12 [95% CI 1.00–1.26]; *p* < 0.05) [40].

### Internal Training Loads

#### Internal Training Load and Physical Qualities

Table 6 presents the relationships between internal training loads and physical qualities. Sixteen studies investigated the relationship between internal training loads and change in physical qualities [34, 41, 46, 47, 49–51, 54, 70, 71, 73, 74, 76, 84, 94, 95]. Of these studies, six found no significant relationships [34, 47, 51, 74, 84].

**Table 6:** Results of relationship between internal training load and change in physical qualities

Monitoring method	Measure	Relationship	References
dRPE	sRPEmus training load vs 15 m	*r* = − 0.15 (90% CL ± 0.39)	[54]
	sRPEmus training load vs 5 m sprint	*r =* − 0.06 (90% CL ± 0.40)	[54]
	sRPEmus training load vs CMJ	*r =* − 0.17 (90% CL ± 0.37)	[54]
	sRPEmus training load vs CMJA	*r =* 0.17 (90% CL ± 0.37)	[54]
	sRPEmus training load vs University of Montreal track test	*r =* 0.69 (90% CL ± 0.20)	[54]
	sRPEres training load vs 15 m sprint	*r =* − 0.21 (90% CL ± 0.39)	[54]
	sRPEres training load vs 5 m sprint	*r =* − 0.02 (90% CL ± 0.41)	[54]
	sRPEres training load vs CMJ	*r =* − 0.06 (90% CL ± 0.38)	[54]
	sRPEres training load vs University of Montreal track test	*r =* 0.71 (90% CL ± 0.19)	[54]
	sRPEres training load vs CMJA	*r =* 0.25 (90% CL ± 0.36)	[54]
Heart rate	bTRIMP vs heart rate at 2 mmol/L—L	*r =* 0.21; *p* > 0.05	[86]
	bTRIMP vs heart rate at 4 mmol/L—L	*r =* − 0.21; *p* > 0.05	[86]
	bTRIMP vs MAS	*r =* 0.03 [95% CI − 0.59 to 0.66]; *R*^2^ = 0.11 [95% CI 0.00 to 0.38]	[92]
	bTRIMP vs velocity at 2 mmol/L	*r =* 0.33 [95% CI − 0.33 to 0.87]; *R*^2^ = 0.23 [95% CI 0.00 to 0.54]	[92]
	bTRIMP vs velocity at 2 mmol/L	*R*^2^ (Quadratic) = 0.31 [99% CI − 0.21 to 0.83]; *p* = 0.26	[34]
	bTRIMP vs velocity at 2 mmol/L	*r =* 0.28; *p* > 0.05	[86]
	bTRIMP vs velocity at 4 mmol/L	*r =* 0.18 [95% CI − 0.48 to 0.81]; *R*^2^ = 0.16 [95% CI 0.00 to 0.46]	[92]
	bTRIMP vs velocity at 4 mmol/L	*R*^2^ (Quadratic) = 0.21 [99% CI − 0.28 to 0.70]; *p* = 0.43	[34]
	bTRIMP vs velocity at 4 mmol/L	*r =* 0.43; *p* > 0.05	[86]
	bTRIMP vs velocity at V̇O_2max_	*R*^2^ (Quadratic) = 0.26 [99% CI − 0.21 to 0.57]; *p* = 0.34	[34]
	bTRIMP vs *V̇*O_2max_	*R*^2^ (Quadratic) = 0.78 [99% CI 0.54 to 1.00]; *p* = 0.005*	[34]
	eTRIMP vs MAS	*r =* 0.09 [95% CI − 0.57 to 0.69]; *R*^2^ = 0.11 [95% CI 0.00 to 0.38]	[92]
	eTRIMP vs MAS	*r =* − 0.21 [90% CI − 0.61 to 0.28]	[51]
	eTRIMP vs velocity at 2 mmol/L	*r =* 0.17 [95% CI − 0.49 to 0.77]; *R*^2^ = 0.13 [95% CI 0.00 to 0.42]	[92]
	eTRIMP vs velocity at 2 mmol/L	*R*^2^ (Quadratic) = 0.11 [99% CI − 0.29 to 0.51]; *p* = 0.65	[34]
	eTRIMP vs velocity at 4 mmol/L	*r =* 0.00 [95% CI − 0.65 to 0.67]; *R*^2^ = 0.10 [95% CI 0.00 to 0.35]	[92]
	eTRIMP vs velocity at 4 mmol/L	*R*^2^ (Quadratic) = 0.27 [99% CI − 0.25 to 0.79]; *p* = 0.34	[34]
	eTRIMP vs velocity at *V̇*O_2max_	*R*^2^ (Quadratic) = 0.02 [99% CI − 0.15 to 0.19]; *p* = 0.93	[34]
	eTRIMP vs *V̇*O_2max_	*R*^2^ (Quadratic) = 0.40 [99% CI − 0.07 to 0.87]; *p* = 0.17	[34]
	eTRIMP vs Yo-yo IR1	*r =* − 0.51	[49]
	iTRIMP vs heart rate at 2 mmol/L—L	*r =* 0.17; *p* > 0.05	[86]
	iTRIMP vs heart rate at 4 mmol/L—L	*r =* − 0.25; *p* > 0.05	[86]
	iTRIMP vs MAS	*r =* 0.37 [95% CI − 0.28 to 0.87]; *R*^2^ = 0.22 [95% CI 0.00 to 0.52]	[92]
	iTRIMP vs velocity at 2 mmol/L	*R*^2^ (Quadratic) = 0.22 [99% CI − 0.29 to 0.72]; *p* = 0.41	[34]
	iTRIMP vs velocity at 2 mmol/L	*r =* 0.93 [95% CI 0.74 to 1]; *R*^2^ = 0.90 [95% CI 0.76 to 0.93]*	[92]
	iTRIMP vs velocity at 2 mmol/L	*r =* 0.67 [95% CI 0.01 to 0.92]; *p* < 0.05*	[86]
	iTRIMP vs velocity at 4 mmol/L	*R*^2^ (Quadratic) = 0.04 [99% CI − 0.20 to 0.28]; *p* = 0.93	[34]
	iTRIMP vs velocity at 4 mmol/L	*r =* 0.88 [95% CI 0.62 to 0.99]; *R*^2^ = 0.82 [95% CI 0.51 to 0.88]*	[92]
	iTRIMP vs velocity at 4 mmol/L	*r =* 0.33; *p* > 0.05	[86]
	iTRIMP vs velocity at V̇O_2max_	*R*^2^ (Quadratic) = 0.15 [99% CI − 0.26 to 0.56]; *p* = 0.56	[34]
	iTRIMP vs *V̇*O_2max_	*R*^2^ (Quadratic) = 0.55 [99% CI 0.09 to 1.00]; *p* = 0.06	[34]
	luTRIMP vs MAS	*r =* 0.26 [95% CI − 0.41 to 0.83]; *R*^2^ = 0.16 [95% CI 0.00 to 0.47]	[92]
	luTRIMP vs velocity at 2 mmol/L	*R*^2^ (Quadratic) = 0.20 [99% CI − 0.29 to 0.53]; *p* = 0.46	[34]
	luTRIMP vs velocity at 2 mmol/L	*r =* 0.75 [95% CI 0.26 to 0.98]; *R*^2^ = 0.60 [95% CI 0.12 to 0.75]*	[92]
	luTRIMP vs velocity at 4 mmol/L	*R*^2^ (Quadratic) = 0.02 [99% CI − 0.16 to 0.21]; *p* = 0.93	[34]
	luTRIMP vs velocity at 4 mmol/L	*r =* 0.82 [95% CI 0.44 to 0.99]; *R*^2^ = 0.69 [95% CI 0.20 to 0.81]*	[92]
	luTRIMP vs velocity at *V̇*O_2max_	*R*^2^ (Quadratic) = 0.49 [99% CI 0.05 to 0.93]; *p* = 0.1	[34]
	luTRIMP vs *V̇*O_2max_	R^2^ (Quadratic) = 0.30 [99% CI − 0.17 to 0.77]; *p* = 0.29	[34]
	Team TRIMP vs heart rate at 2 mmol/L—L	*r =* 0.28; *p* > 0.05	[86]
	Team TRIMP vs heart rate at 4 mmol/L—L	*r =* − 0.49; *p* > 0.05	[86]
	Team TRIMP vs velocity at 2 mmol/L—L	*r =* 0.20; *p* > 0.05	[86]
	Team TRIMP vs velocity at 4 mmol/L—L	*r =* 0.28; *p* > 0.05	[86]
sRPE	1 week training load vs anaerobic sprint rest average power	*r =* − 0.04; *p* > 0.05	[73]
	1 week training load vs anaerobic sprint test fatigue index	*r =* 0.32; *p* > 0.05	[73]
	1 week training load vs anaerobic sprint test minimum power	*r =* 0.11; *p* > 0.05	[73]
	1 week training load vs anaerobic sprint test peak power	*r =* − 0.08; *p* > 0.05	[73]
	1 week training load vs change of direction	*r =* 0.38; *p* > 0.05	[73]
	1 week training load vs Yo-yo IR1	*r =* − 0.07	[49]
	4-week ACWR vs anaerobic sprint rest average power	*r =* 0.13; *p* > 0.05	[73]
	4-week ACWR vs anaerobic sprint test fatigue index	*r =* 0.04; *p* > 0.05	[73]
	4-week ACWR vs anaerobic sprint test minimum power	*r =* − 0.05; *p* > 0.05	[73]
	4-week ACWR vs anaerobic sprint test peak power	*r =* 0.08; *p* > 0.05	[73]
	4-week ACWR vs change of direction	*r =* 0.45; *p* < 0.05*	[73]
	Chronic workload vs anaerobic sprint rest average power	*r =* 0.09; *p* > 0.05	[73]
	Chronic workload vs anaerobic sprint test fatigue index	*r =* − 0.22; *p* > 0.05	[73]
	Chronic workload vs anaerobic sprint test minimum power	*r =* − 0.01; *p* > 0.05	[73]
	Chronic workload vs anaerobic sprint test peak power	*r =* 0.09; *p* > 0.05	[73]
	Chronic workload vs change of direction	*r =* − 0.43; *p* < 0.05*	[73]
	Aerobic conditioning training load vs 10 m sprint	*r =* − 0.47; R^2^ = 0.22	[46]
	Aerobic conditioning training load vs 10 m sprint momentum	*r =* 0.51; R^2^ = 0.26	[46]
	Aerobic conditioning training load vs 20 m sprint	*r =* − 0.65; R^2^ = 0.42	[46]
	Aerobic conditioning training load vs 20 m sprint momentum	*r =* 0.52; R^2^ = 0.28	[46]
	Aerobic conditioning training load vs change of direction	*r =* 0.14; R^2^ = 0.02	[46]
	Aerobic conditioning training load vs CMJ	*r =* 0.19; R^2^ = 0.03	[46]
	Aerobic conditioning training load vs power pass	*r =* 0.03; R^2^ = 0.01	[46]
	Aerobic conditioning training load vs prone Yo-Yo IR1	*r =* 0.01; R^2^ = 0.00	[46]
	Intensification period vs CMJ	*g* = 0.11 [90% CI − 0.37 to 0.59]	[36]
	Intensification period vs left hip flexibility	*g* = − 0.11 [90% CI − 0.59 to 0.85]	[36]
	Intensification period vs push ups	*g* = − 0.03 [90% CI − 0.51 to 0.46]	[36]
	Intensification period vs right hip flexibility	*g* = 0.07 [90% CI − 0.7 to 0.49]	[36]
	Intensification period vs sit ups	*g* = 0.13 [90% CI − 0.36 to 0.61]	[36]
	Monotony vs anaerobic sprint rest average power	*r =* 0.08; *p* > 0.05	[73]
	Monotony vs anaerobic sprint test fatigue index	*r =* − 0.1; *p* > 0.05	[73]
	Monotony vs anaerobic sprint test minimum power	*r =* − 0.15; *p* > 0.05	[73]
	Monotony vs anaerobic sprint test peak power	*r =* 0.08; *p* > 0.05	[73]
	Monotony vs change of direction	*r =* − 0.17; *p* > 0.05	[73]
	Monotony vs lactate minimum speed (competitive period)	*ρ* = − 0.31; *p* > 0.05	[41]
	Monotony vs lactate minimum speed (general period)	*ρ* = 0.51; *p* > 0.05	[41]
	Monotony vs lactate minimum speed (specific period)	*ρ* = 0.14; *p* > 0.05	[41]
	Monotony vs repeated sprint ability (competition period)	*ρ* = − 0.63; *p* < 0.05*	[41]
	Monotony vs repeated sprint ability (competition period)	*ρ* = − 0.52; *p* < 0.05*	[41]
	Monotony vs repeated sprint ability (general period)	*ρ* = − 0.17; *p* > 0.05	[41]
	Monotony vs repeated sprint ability (specific period)	*ρ* = − 0.36; *p* > 0.05	[41]
	Monotony vs repeated sprint ability (specific period)	*ρ* = − 0.58; *p* < 0.05*	[41]
	Monotony vs repeated sprint ability (general period)	*ρ* = − 0.16; *p* > 0.05	[41]
	On-court training load on tour vs 10 m sprint	*r =* 0.45; *p* ≤ 0.05*	[70]
	On-court training load on tour vs 10 × 20 m repeated sprint ability	*r =* 0.27; *p* > 0.05	[70]
	On-court training load on tour vs 20 m sprint	*r =* 0.52; *p* ≤ 0.05*	[70]
	On-court training load on tour vs 5-0-5 left	*r =* 0.24; *p* > 0.05	[70]
	On-court training load on tour vs 5-0-5 right	*r =* 0.09; *p* > 0.05	[70]
	On-court training load on tour vs 5 m sprint	*r =* 0.26; *p* > 0.05	[70]
	On-court training load on tour vs CMJ	*r =* 0.04; *p* > 0.05	[70]
	On-court training load on tour vs multi-stage fitness test	*r =* –0.48; *p* ≤ 0.05*	[70]
	On-court training load on tour vs single leg CMJ (dominant)	*r =* –0.06; *p* > 0.05	[70]
	On-court training load on tour vs single leg CMJ (non-dominant)	*r =* –0.06; *p* > 0.05	[70]
	On-court training load pre-tour vs 10 m sprint	*r =* –0.07; *p* > 0.05	[70]
	On-court training load pre-tour vs 10 × 20 m repeated sprint ability	*r =* –0.37; *p* ≤ 0.05*	[70]
	On-court training load pre-tour vs 20 m sprint	*r =* –0.13; *p* > 0.05	[70]
	On-court training load pre-tour vs 5-0-5 left	*r =* 0.25; *p* > 0.05	[70]
	On-court training load pre-tour vs 5-0-5 right	*r =* 0.16; *p* > 0.05	[70]
	On-court training load pre-tour vs 5 m sprint	*r =* –0.10; *p* > 0.05	[70]
	On-court training load pre-tour vs CMJ	*r =* 0.40; p ≤ 0.05*	[70]
	On-court training load pre-tour vs multi-stage fitness test	*r =* –0.19; *p* > 0.05	[70]
	On-court training load pre-tour vs single-leg CMJ (dominant)	*r =* 0.16; *p* > 0.05	[70]
	On-court training load pre-tour vs single-leg CMJ (non-dominant)	*r =* 0.07; *p* > 0.05	[70]
	Resistance training load vs 10 m sprint	*r =* − 0.52; R^2^ = 0.273	[46]
	Resistance training load vs 10 m sprint momentum	*r =* 0.12; R^2^ = 0.014	[46]
	Resistance training load vs 20 m sprint	*r =* − 0.49; R^2^ = 0.236	[46]
	Resistance training load vs 20 m sprint momentum	*r =* 0.01; R^2^ = 0	[46]
	Resistance training load vs change of direction	*r =* 0.42; R^2^ = 0.18	[46]
	Resistance training load vs CMJ	*r =* 0.51; R^2^ = 0.26	[46]
	Resistance training load vs power pass	*r =* 0.40; R^2^ = 0.16	[46]
	Resistance training load vs prone Yo-Yo IR1	*r =* 0.04; R^2^ = 0.01	[46]
	S&C training load on tour vs 10 m sprint	*r =* –0.07; *p* > 0.05	[70]
	S&C training load on tour vs 10 × 20 m repeated sprint ability	*r =* 0.36; *p* ≤ 0.05*	[70]
	S&C training load on tour vs 20 m sprint	*r =* –0.08; *p* > 0.05	[70]
	S&C training load on tour vs 5-0-5 left	*r =* 0.01; *p* > 0.05	[70]
	S&C training load on tour vs 5-0-5 right	*r =* 0.01; *p* > 0.05	[70]
	S&C training load on tour vs 5 m sprint	*r =* 0.27; *p* > 0.05	[70]
	S&C training load on tour vs CMJ	*r =* –0.19; *p* > 0.05	[70]
	S&C training load on tour vs multi-stage fitness test	*r =* –0.04; *p* > 0.05	[70]
	S&C training load on tour vs single-leg CMJ (dominant)	*r =* –0.12; *p* > 0.05	[70]
	S&C training load on tour vs single-leg CMJ (non-dominant)	*r =* 0.28; *p* > 0.05	[70]
	S&C training load pre-tour vs 10 m sprint	*r =* –0.11; *p* > 0.05	[70]
	S&C training load pre-tour vs 10 × 20 m repeated sprint ability	*r =* –0.11; *p* > 0.05	[70]
	S&C training load pre-tour vs 20 m sprint	*r =* –0.09; *p* > 0.05	[70]
	S&C training load pre-tour vs 5-0-5 left	*r =* 0.25; *p* > 0.05	[70]
	S&C training load pre-tour vs 5-0-5 right	*r =* 0.32; *p* > 0.05	[70]
	S&C training load pre-tour vs 5 m sprint	*r =* –0.06; *p* > 0.05	[70]
	S&C training load pre-tour vs CMJ	*r =* 0.03; *p* > 0.05	[70]
	S&C training load pre-tour vs multi-stage fitness test	*r =* –0.02; *p* > 0.05	[70]
	S&C training load pre-tour vs single-leg CMJ (dominant)	*r =* 0.1; *p* > 0.05	[70]
	S&C training load pre-tour vs single-leg CMJ (non-dominant)	*r =* 0.06; *p* > 0.05	[70]
	Skill training load vs 10 m sprint	*r =* − 0.71; R^2^ = 0.51	[46]
	Skill training load vs 10 m sprint momentum	*r =* 0.35; R^2^ = 0.12	[46]
	Skill training load vs 20 m sprint	*r =* − 0.79; R^2^ = 0.62	[46]
	Skill training load vs 20 m sprint momentum	*r =* 0.27; R^2^ = 0.07	[46]
	Skill training load vs change of direction	*r =* 0.20; R^2^ = 0.04	[46]
	Skill training load vs CMJ	*r =* 0.60; R^2^ = 0.36	[46]
	Skill training load vs power pass	*r =* 0.22; R^2^ = 0.05	[46]
	Skill training load vs prone Yo-Yo IR1	*r =* 0.11; R^2^ = 0.01	[46]
	Skill training load vs prone Yo-Yo IR1	*r =* 0.11; R^2^ = 0.01	[46]
	Strain vs anaerobic sprint rest average power	*r =* − 0.10; *p* > 0.05	[73]
	Strain vs anaerobic sprint test fatigue index	*r =* 0.35; *p* > 0.05	[73]
	Strain vs anaerobic sprint test minimum power	*r =* 0.18; *p* > 0.05	[73]
	Strain vs anaerobic sprint test peak power	*r =* − 0.13; *p* > 0.05	[73]
	Strain vs change of direction	*r =* 0.42; *p* < 0.05*	[73]
	Strain vs lactate minimum speed (competitive period)	*ρ* = − 0.36; *p* > 0.05	[41]
	Strain vs lactate minimum speed (general period)	*ρ* = 0.42: *p* > 0.05	[41]
	Strain vs lactate minimum speed (specific period)	*ρ* = 0.07; *p* > 0.05	[41]
	Strain vs repeated sprint ability (competition period)	*ρ* = − 0.42 *p* > 0.05	[41]
	Strain vs repeated sprint ability (competition period)	*ρ* = 0.53; *p* < 0.05*	[41]
	Strain vs repeated sprint ability (general period)	*ρ* = − 0.10; *p* > 0.05	[41]
	Strain vs repeated sprint ability (general period)	*ρ* = 0.12; *p* > 0.05	[41]
	Strain vs repeated sprint ability (specific period)	*ρ* = 0.37; *p* > 0.05	[41]
	Strain vs repeated sprint ability (specific period)	*ρ* = − 0.34; *p* > 0.05	[41]
	Sum of perceived exertion Under 15 vs 15 m sprint	*r =* 0.57 (90% CI ± 0.48)	[76]
	Sum of perceived exertion Under 15 vs 5 m sprint	*r =* 0.67 (90% CI ± 42)	[76]
	Sum of perceived exertion Under 15 vs CMJ	*r =* − 0.70 (90% CI ± 0.4)	[76]
	Sum of perceived exertion Under 15 vs T-Test	*r =* 0.53 (90% CI ± 0.51)	[76]
	Sum of perceived exertion Under 15 vs Yo-Yo IR1	*r =* − 0.78 (90% CI ± 0.32	[76]
	Sum of perceived exertion Under 16 vs 15 m sprint	*r =* 0.44 (90% CI ± 0.47)	[76]
	Sum of perceived exertion Under 16 vs 5 m sprint	*r =* 0.47 (90% CI ± 0.47)	[76]
	Sum of perceived exertion Under 16 vs CMJ	*r =* 0.39 (90% CI ± 0.49)	[76]
	Sum of perceived exertion Under 16 vs T-Test	*r =* 0.11 (90% CI ± 0.55)	[76]
	Sum of perceived exertion Under 16 vs Yo-Yo IR1	*r =* 0.22 (90% CI ± 0.51)	[76]
	Taper period vs CMJ	*g* = − 0.11 [90% CI − 0.58 to 0.38]	[36]
	Taper period vs left hip flexibility	*g* = 0.42 [90% CI − 0.39 to 1.23]	[36]
	Taper period vs push ups	*g* = 0.61 [90% CI 1.09 to 0.11] (sic)	[36]
	Taper period vs right hip flexibility	*g* = 0.24 [90% CI − 0.54 to 1.02]	[36]
	Taper period vs sit ups	*g* = 0.8 [90% CI 0.29 to 1.29]*	[36]
	Total tennis training load vs 10 m sprint	*r =* 0.45	[70]
	Total tennis training load vs 20 m sprint	*r =* 0.52	[70]
	Total tennis training load vs multi-stage fitness test	*r =* − 0.44	[70]
	Total training load vs change of direction	*r =* 0.32; R^2^ = 0.105	[46]
	Total training load vs CMJ	*r =* 0.55; R^2^ = 0.306	[46]
	Total training load vs power pass	*r =* 0.29; R^2^ = 0.084	[46]
	Training load in overload period vs Yo-Yo IR1	*d* = − 1.48 [0/0/100]; *p* < 0.016	[50]
	Training load in taper vs Yo-Yo IR1	*d* = 1.83 [100/0/0]; *p* < 0.016	[50]
	Training load on tour vs 10 m sprint	*r =* 0.38; *p* ≤ 0.05*	[70]
	Training load on tour vs 10 × 20 m repeated sprint ability	*r =* 0.36; *p* > 0.05	[70]
	Training load on tour vs 20 m sprint	*r =* 0.44; *p* ≤ 0.05*	[70]
	Training load on tour vs 5–0-5 left	*r =* 0.22; *p* > 0.05	[70]
	Training load on tour vs 5–0-5 right	*r =* 0.08; *p* > 0.05	[70]
	Training load on tour vs 5 m sprint	*r =* 0.31; *p* > 0.05	[70]
	Training load on tour vs CMJ	*r =* –0.02; *p* > 0.05	[70]
	Training load on tour vs multi-stage fitness test	*r =* –0.40; *p* ≤ 0.05*	[70]
	Training load on tour vs single-leg CMJ (dominant)	*r =* –0.09; *p* > 0.05	[70]
	Training load on tour vs single-leg CMJ (non-dominant)	*r =* 0.03; *p* > 0.05	[70]
	Training load pre-tour vs 10 m sprint	*r =* –0.08; *p* > 0.05	[70]
	Training load pre-tour vs 10 × 20 m repeated sprint ability	*r =* –0.36; *p* > 0.05	[70]
	Training load pre-tour vs 20 m sprint	*r =* –0.14; *p* > 0.05	[70]
	Training load pre-tour vs 5–0-5 left	*r =* 0.27; *p* > 0.05	[70]
	Training load pre-tour vs 5–0-5 right	*r =* 0.17; *p* > 0.05	[70]
	Training load pre-tour vs 5 m sprint	*r =* –0.10; *p* > 0.05	[70]
	Training load pre-tour vs CMJ	*r =* 0.38; *p* ≤ 0.05*	[70]
	Training load pre-tour vs multi-stage fitness test	*r =* –0.18; *p* > 0.05	[70]
	Training load pre-tour vs single-leg CMJ (dominant)	*r =* 0.17; *p* > 0.05	[70]
	Training load pre-tour vs single-leg CMJ (non-dominant)	*r =* 0.07; *p* > 0.05	[70]
	Training load Under 15 vs 15 m sprint	*r =* 0.55 (90% CI ± 0.5)	[76]
	Training load Under 15 vs 5 m sprint	*r =* 0.64 (90% CI ± 0.44)	[76]
	Training load Under 15 vs CMJ	*r =* − 0.65 (90% CI ± 0.43)	[76]
	Training load Under 15 vs T-Test	*r =* 0.52 (90% CI ± 0.51)	[76]
	Training load Under 15 vs Yo-Yo IR1	*r =* − 0.78 (90% CI ± 0.32)	[76]
	Training load Under 16 vs 15 m sprint	*r =* 0.42 (90% CI ± 0.48)	[76]
	Training load Under 16 vs 5 m sprint	*r =* 0.45 (90% CI ± 0.47)	[76]
	Training load Under 16 vs CMJ	*r =* 0.39 (90% CI ± 0.49)	[76]
	Training load Under 16 vs T-Test	*r =* 0.10 (90% CI ± 0.55)	[76]
	Training load Under 16 vs Yo-Yo IR1	*r =* 0.22 (90% CI ± 0.51)	[76]
	Training load vs 10 m sprint	*r =* − 0.70; R^2^ = 0.488	[46]
	Training load vs 10 m sprint	*p* = 0.70	[73]
	Training load vs 10 m sprint momentum	*r =* 0.36; R^2^ = 0.13	[46]
	Training load vs 20 m sprint	*r =* − 0.77; R^2^ = 0.60	[46]
	Training load vs 20 m sprint momentum	*r =* 0.29; R^2^ = 0.08	[46]
	Training load vs 30 m sprint	*p* = 0.51	[73]
	Training load vs anaerobic sprint rest average power	*p* = 0.93	[73]
	Training load vs anaerobic sprint test fatigue index	*p* = 0.67	[73]
	Training load vs anaerobic sprint test minimum power	*p* = 0.23	[73]
	Training load vs anaerobic sprint test peak power	*p* = 0.34	[73]
	Training load vs change in MAS	*r =* 0.37 [95% CI − 0.27 to 0.88]; *R*^2^ = 0.24 [0.00 − 0.55]	[92]
	Training load vs change in velocity at 2 mmol/L	*r =* − 0.17 [95% CI − 0.77 to 0.50]; *R*^2^ = 0.12 [0.00–0.40]	[92]
	Training load vs change in velocity at 4 mmol/L	*r =* − 0.16 [95% CI − 0.76 to 0.51]; *R*^2^ = 0.12 [0.00–0.39]	[92]
	Training load vs CMJ	*d* = − 0.9	[84]
	Training load vs heart rate at 2 mmol/L—L	*r =* 0.20; *p* > 0.05	[34]
	Training load vs heart rate at 4 mmol/L—L	*r =* 0.15; *p* > 0.05	[34]
	Training load vs lactate minimum speed (competitive period)	*ρ* = − 0.18; *p* > 0.05	[41]
	Training load vs lactate minimum speed (general period)	*ρ* = 0.55; *p* < 0.05*	[41]
	Training load vs lactate minimum speed (general period)	*ρ* = 0.01; *p* > 0.05	[41]
	Training load vs lactate minimum speed (specific period)	*ρ* = − 0.10; *p* > 0.05	[41]
	Training load vs MAS	*r =* 0.22 [90% CI − 0.26 to 0.62]	[51]
	Training load vs modified 5-0-5	*p* = 0.16	[73]
	Training load vs MSS	*r =* 0.37 [90% CI − 0.11 to 0.71]	[51]
	Training load vs prone Yo-Yo IR1	*r =* 0.07; R^2^ = 0.005	[46]
	Training load vs repeated sprint ability (competition period)	*ρ* = 0.35; *p* > 0.05	[41]
	Training load vs repeated sprint ability (competition period)	*ρ* = − 0.26; *p* > 0.05	[41]
	Training load vs repeated sprint ability (general period)	*ρ* = 0.12; *p* > 0.05	[41]
	Training load vs repeated sprint ability (general period)	*ρ* = 0.02; *p* > 0.05	[41]
	Training load vs repeated sprint ability (specific period)	*ρ* = − 0.18; *p* > 0.05	[41]
	Training load vs repeated sprint ability (specific period)	*ρ* = − 0.12; *p* > 0.05	[41]
	Training load vs velocity at 2 mmol/L	*R =* 0.11 [99% CI − 0.29 to 0.51]; *p* = 0.66	[86]
	Training load vs velocity at 2 mmol/L—L	*r =* 0.13; *p* > 0.05	[34]
	Training load vs velocity at 4 mmol/L	*R =* 0.07 [99% CI − 0.13 to 0.27]; *p* = 0.77	[86]
	Training load vs velocity at 4 mmol/L—L	*r =* 0.40; *p* > 0.05	[34]
	Training load vs velocity at *V̇*O_2max_	*R =* 0.14 [99% CI − 0.26 to 0.54]; *p* = 0.59	[86]
	Training load vs *V̇*O_2max_	*R* = 0.12 [99% CI − 0.30 to 0.54]; *p* = 0.65	[86]

*ACWR* acute to chronic work ratio, *bTRIMP* Banister’s training impulse, *CMJ* countermovement jump, *CMJA* countermovement jump with arm swing, *dRPE* differential rating of perceived exertion, *eTRIMP* Edwards’ training impulse, *iTRIMP* individual training impulse, *luTRIMP* Lucia’s training impulse, *MAS* maximal aerobic speed, *MSS* maximal sprint speed, *sRPE* session ratings of perceived exertion, *sRPEmus* session ratings of perceived exertion muscular, *sRPEres* session ratings of perceived exertion respiratory, *S&C* strength and conditioning, *TeamTRIMP* team training impulse

^*^Statistically significant result

Heart rate metrics had inconsistent or limited evidence of a relationship to changes in physical qualities. Positive relationships with aerobic fitness were observed for individualised training impulse (iTRIMP) [47, 94], while Banister’s training impulse (bTRIMP), Lucia’s training impulse (LuTRIMP), and Edwards’ training impulse (eTRIMP) all had both non-significant and positive relationships observed [34, 47, 49, 51, 94]. Maximal sprint speed was also found to have a positive relationship with eTRIMP [94], although the strength of the evidence was limited.

The evidence of a relationship between sRPE and physical qualities was inconsistent or limited. There were non-significant [34, 46, 47, 86], positive [41, 49], and negative [50, 70, 76] findings for aerobic fitness; negative [70, 71, 76] and positive [46] findings for speed; negative [46, 76] and non-significant [70] findings for change of direction ability; non-significant findings for flexibility [95]; negative findings for muscular endurance [95]; and non-significant [41, 73, 74] findings for repeated sprint ability.

Studies investigating differential ratings of perceived exertion (dRPE) were limited, with various methods of quantifying load and inconsistent results. A positive relationship was seen between dRPE and aerobic fitness, but there were non-significant findings for speed and power [54]. Relationships between aerobic conditioning training load and physical qualities were negative for speed [46], and non-significant for power, change of direction, or aerobic fitness [46]. Tactical or skill-based training load showed both non-significant [46] and negative [70, 71] relationships with aerobic fitness and negative relationships with repeated sprint ability [70]. A positive relationship was observed between strength and conditioning load, determined by the sRPE from all off-court training including resistance and metabolic conditioning, and repeated sprint ability, but there were non-significant results for speed, change of direction, aerobic fitness, and power [70]. Resistance training load showed positive relationships with speed, change of direction, and power [46].

#### Internal Training Loads and Injury

Table 7 presents the relationships between internal training loads and injury. Ten studies found significant relationships between internal training load and injury [33, 40, 45, 63, 69, 72, 75, 77, 83, 90], whilst one found no relationship [58]. Studies used a number of different definitions of injury, including reporting of a physical complaint or medical attention [33, 40, 69], time-loss injuries [45, 63, 72, 75, 83, 90], and time loss > 3 weeks [58]. However, when pooling all the contributing findings from included studies, only 25% of contributing findings showed a relationship between internal training loads and injury.

**Table 7:** Results of relationship between internal training load and change in injury risk

Monitoring method	Measure vs injury risk	Relationship	References
Heart rate	eTRIMP	1 Unit = increase in injury risk; *p* = 0.014*	[81]
Novel scale	Annual high intensity	*p* = 0.06	[58]
	Annual training load	*p* = 0.10	[58]
	Average hours	*p* = 0.36	[58]
	Total high intensity	*p* = 0.16	[58]
	Total training hours	*p* = 0.54	[58]
	Total training load	*p* = 0.24	[58]
sRPE	1-week load	RR = 1.11 [95% CI 0.84–1.50]; *p* = 0.44	[45]
	1-week load	OR = 1.00 [90% CI 0.99–1.00]	[83]
	1-week load	OR = 0.56 [95% CI 0.42–0.73]; *p* < 0.001*	[63]
	1-week load	OR = 1.43 [95% CI 1.07–1.92]; *p* = 0.015*	[63]
	1-week differential load	*p* = 0.86	[77]
	1-week EWMA load	RR = 1.88 [95% CI 1.21–1.91]; *p* = 0.005	[77]
	1-week load > 898 AU	OR = 2.75 [95% CI 1.00–7.59]; *p* = 0.05*	[75]
	1-week load > 6844 AU (< 3330 reference)	RR = 2.12 [95% CI 0.77–5.85]	[69]
	1-week load > 6844 AU (3330–4994 reference)	RR = 1.93 [95% CI 0.90–4.15]	[69]
	1-week load > 6844 AU (4995–6844 reference)	RR = 2.29 [95% CI 1.03–5.07]*	[69]
	1-week load 3330–4994 AU (< 3330 reference)	RR = 1.10 [95% CI 0.40–2.98]	[69]
	1-week load 4995–6844 AU (< 3330 reference)	RR = 0.93 [95% CI 0.33–2.59]	[69]
	1-week load 4995–6844 AU (3330–4994 reference)	RR = 0.85 [95% CI 0.39–1.84]	[69]
	1-week load	OR = 1.62 [CI 1.16–2.29]; *p* = 0.005*	[90]
	1-week load vs overuse injury	OR = 1.01 [95% CI 1.00–1.02]; *p* ≥ 0.05	[40]
	1-week load vs traumatic injury	OR = 1.01 [95% CI 1.00–1.02]; *p* < 0.05*	[40]
	2-week ACWR	RR = 0.99 [95% CI 0.90–1.09]; *p* = 0.82	[45]
	2-week load	RR = 1.03 [95% CI 0.77–1.38]; *p* = 0.85	[45]
	2-week load	OR = 1.01 [95% CI 0.91–1.11]; *p* = 0.90	[63]
	2-week training load > 1713 AU	OR = 2.57 [95% CI 0.94–7.07]; *p* = 0.07	[75]
	3-week ACWR	RR = 1.00 [95% CI 0.95–1.06]; *p* = 0.91	[45]
	3-week load	RR = 0.97 [95% CI 0.74–1.28]; *p* = 0.82	[45]
	3-week load	OR = 0.99 [95% CI 0.89–1.11]; *p* = 0.90	[63]
	3-week training load > 2376 AU	OR = 2.57 [95% CI 0.94–7.07]; *p* = 0.07	[75]
	4-week ACWR	RR = 1.01 [95% CI 0.96–1.07]; *p* = 0.73	[45]
	4-week ACWR	HR = 2.76 [95% CI 1.58–4.82]; *p* < 0.01*	[72]
	4-week ACWR	OR = 0.16 [90% CI 0.01–1.84]	[83]
	4-week ACWR	OR = 1.20 [95% CI 0.87–1.64]; *p* = 0.26	[63]
	4-week ACWR	OR = 0.68 [95% CI 0.40–0.96]; *p* = 0.03*	[63]
	4-week ACWR > 1.3	OR = 0.40 [95% CI 0.13–1.22]; *p* = 0.11	[75]
	4-week ACWR vs injury	OR = 1.59 [CI 1.1–2.5]; *p* = 0.03*	[90]
	4-week load	RR = 1.00 [95% CI 0.76–1.33]; *p* = 0.97	[45]
	4-week load	OR = 0.92 [95% CI 0.83–1.03]; *p* = 0.13	[63]
	4-week load > 2996 AU	OR = 2.57 [95% CI 0.94–7.07]; *p* = 0.07	[75]
	4-week load	OR = 1.13 [CI 0.75–1.67]; *p* = 0.55	[90]
	Daily training load	OR = 1.98 [CI 1.43–2.78]; *p* < 0.01*	[90]
	Daily training load	OR = 1.91 [CI 1.40–2.63]; *p* < 0.01*	[90]
	High (> 0.35) 3-day training load z-score	RR = 2.4 [1.57–3.66]; *p* < 0.001*	[33]
	High (> 0.67) 14-day training load z-score	RR = 1.89 [1.26–2.85]; *p* = 0.01*	[33]
	High (> 1.30) EWMA ACWR	RR = 1.01 [0.65–1.58]; Unclear; *p* = 0.96	[33]
	High 1-week training load	RR = 1.65; *p* < 0.05	[38]
	High 2-week training load	RR = 1.03; *p* > 0.05	[38]
	High 3-week training load	RR = 1.09; *p* > 0.05	[38]
	High 4-week training load	RR = 1.2; *p* > 0.05	[38]
	High 4-week training load ACWR	RR = 1.01; *p* > 0.05	[38]
	High 4-week training load ACWR with high chronic workload	RR = 0.43; *p* > 0.05	[38]
	High 4-week training load ACWR with low chronic workload	RR = 1.59; *p* > 0.05	[38]
	Low 1-week training load	RR = 0.27; *p* < 0.05	[38]
	Low 2-week training load	RR = 0.5; *p* > 0.05	[38]
	Low 3-week training load	RR = 0.55; *p* > 0.05	[38]
	Low 4-week training load	RR = 0.75; *p* > 0.05	[38]
	Low 4-week training load ACWR	RR = 0.84; *p* > 0.05	[38]
	Low 4-week training load ACWR with high chronic workload	RR = 0.81; *p* > 0.05	[38]
	Low 4-week training load ACWR with low chronic workload	RR = 0.37; *p* > 0.05	[38]
	Medium (< 0.45–0.35) 3-day training load z-score	RR = 1.18 [0.73–1.93]; *p* = 0.56	[33]
	Medium (− 0.40 to 0.67) 14-day training load z-score	RR = 1.18 [0.82–1.71]; *p* = 0.46	[33]
	Medium (0.80–1.30) EWMA ACWR	RR = 0.99 [0.64–1.56]; *p* = 0.99	[33]
	Moderate–high 1-week training load	RR = 0.98; *p* > 0.05	[38]
	Moderate–high 2-week training load	RR = 1.38; *p* > 0.05	[38]
	Moderate–high 3-week training load	RR = 1.39; *p* > 0.05	[38]
	Moderate–high 4-week training load	RR = 1.12; *p* > 0.05	[38]
	Moderate–high 4-week training load ACWR	RR = 1.34; *p* > 0.05	[38]
	Moderate–high 4-week training load ACWR with high chronic workload	RR = 1.34; *p* > 0.05	[38]
	Moderate–high 4-week training load ACWR with low chronic workload	RR = 1.16; *p* > 0.05	[38]
	Moderate–low 1-week training load	RR = 1.45; *p* > 0.05	[38]
	Moderate–low 2-week training load	RR = 1.07; *p* > 0.05	[38]
	Moderate–low 3-week training load	RR = 0.98; *p* > 0.05	[38]
	Moderate–low 4-week training load	RR = 1.01; *p* > 0.05	[38]
	Moderate–low 4-week training load ACWR	RR = 1.15; *p* > 0.05	[38]
	Moderate–low 4-week training load ACWR with high chronic workload	RR = 1.22; *p* > 0.05	[38]
	Moderate–low 4-week training load ACWR with low chronic workload	RR = 1.15; *p* > 0.05	[38]
	Monotony	OR = 1.01 [95% CI 0.92–1.11]; *p* = 0.843	[63]
	Monotony > 0.53	OR = 6.16 [95% CI 1.58–24.06]; *p* = 0.01*	[75]
	Monotony > 0.53	OR = 4.17 [95% CI 1.48–11.72]; *p* = 0.01*	[75]
	Monotony vs overuse injury	OR = 0.84 [95% CI 0.25–2.76]; *p* ≥ 0.05	[40]
	Monotony vs traumatic injury	OR = 2.59 [95% CI 1.22–5.50]; *p* < 0.05*	[40]
	Prior-day training load vs injury	OR = 1.38 [CI 1.01–1.88]; *p* = 0.040*	[90]
	Prior-day training load	OR = 1.42 [CI 1.04–1.95]; *p* = 0.027*	[90]
	Session load	OR = 0.64 [95% CI 0.49–0.83] p < 0.01*	[63]
	Session load	OR = 1.44 [95% CI 1.11–1.88]; *p* < 0.01*	[63]
	Strain	OR = 0.63 [95% CI 0.45–0.88]; *p* < 0.01*	[63]
	Strain > 809 AU	OR = 0.35 [95% CI 0.05–2.32]; *p* = 0.28	[75]
	Strain	OR = 1.41 [95% CI 1.02–1.93]; *p* = 0.03*	[63]
	Strain > 809 AU	OR = 2.49 [95% CI 0.79–7.88]; *p* = 0.12	[75]
	Strain vs overuse injury	OR = 1.00 [95% CI 1.00–1.01]; *p* ≥ 0.05	[40]
	Strain vs traumatic injury	OR = 1.01 [95% CI 1.00–1.01]; *p* < 0.05*	[40]
	Very high 1-week training load	RR = 2; *p* > 0.05	[38]
	Very high 2-week training load	RR = 1.93; *p* > 0.05	[38]
	Very high 3-week training load	RR = 1.59; *p* > 0.05	[38]
	Very high 4-week training load	RR = 1.84; *p* > 0.05	[38]
	Very high 4-week training load ACWR	RR = 1.17; *p* > 0.05	[38]
	Very high 4-week training load ACWR with high chronic workload	RR = 2.67; *p* > 0.05	[38]
	Weekly change in load	RR = 1.00 [95% CI 0.96–1.04]; *p* = 0.93	[45]
	Weekly change in load	OR = 1.00 [95% CI 0.93–1.07]; *p* = 0.95	[63]
	Weekly change in load > 410 AU	OR = 3.70 [95% CI 0.87–15.75]; *p* = 0.41	[75]
	Weekly change in load > 410 AU	OR = 3.27 [95% CI 1.15–9.32]; *p* = 0.03*	[75]
	Weekly percentage change in load	OR = 0.94 [95% CI 0.86–1.03]; *p* = 0.21	[63]

*ACWR* acute to chronic work to rest ratio, *AU* arbitrary units, *eTRIMP* Edwards’ training impulse, *EWMA* exponentially weighted moving average, *OR* odds ratio, *RR* relative risk

^*^Statistically significant result

The evidence of a relationship between sRPE and injury risk was limited. There were positive [40, 69, 75, 90], non-significant [45, 83], and variable [63, 77] relationships between 1-week sRPE and injury risk. Two-week training load and injury had positive [33], and non-significant [33, 63, 75] results. No significant relationship was seen for 3- and 4-week training load, annual high-intensity training load, or annual training load and injury risk [45, 63, 75]. Daily training load [90], prior day’s training load [90], and individual sessional load [63] were all found to be positively related to injury risk.

Some studies investigated the change in training loads using statistical methods such as the acute to chronic work ratio (ACWR), monotony, and strain. These alternative methods of analysing internal training loads had inconsistent relationships with injury risk. Results were non-significant [33, 45, 63, 72, 75, 83, 90] and positive [59, 60, 63, 72] for ACWR; and non-significant [40, 63, 75] and positive [40, 75] for strain and monotony.

#### Internal Training Loads and Illness

Table 8 presents the relationships between internal training loads and illness. Seven studies investigated the relationship between internal training load and illness [40–42, 53, 90, 95]. Both non-significant [40–42, 53, 95] and positive [90] relationships were reported for sRPE. The only study that investigated the relationship between HR and injury risk found a positive relationship [81].

**Table 8:** Results of relationship between internal training load and illness

Monitoring method	Measure	Relationship	References
sRPE	Intensification and taper periods vs URTI symptoms	χ^2^ = 2.81; *p* = 0.24	[36]
	1-week load vs illness	OR = 1.00 [95% CI 0.99–1.02]; *p* ≥ 0.05	[40]
	Monotony vs illness	OR = 2.52 [95% CI 0.79–8.08]; *p* ≥ 0.05	[40]
	Strain vs illness	OR = 1.00 [95% CI 1.00–1.01]; *p* ≥ 0.05	[40]
	Training load vs URTI incidence (Period 1)	*ρ* = 0.09; *p* > 0.05	[41]
	Training load vs URTI incidence (Period 2)	*ρ* = − 0.20; *p* > 0.05	[41]
	Training load vs URTI incidence (Period 3)	*ρ* = − 0.19; *p* > 0.05	[41]
	Training load vs URTI severity (Period 1)	*ρ* = − 0.07; *p* > 0.05	[41]
	Training load vs URTI severity (Period 2)	*ρ* = − 0.15; *p* > 0.05	[41]
	Training load vs URTI severity (Period 3)	*ρ* = 0.06; *p* > 0.05	[41]
	Week 1 Weekly load vs URTI symptoms	*r* = 0.3; *p* = 0.34	[42]
	Week 2 Weekly load vs URTI symptoms	*r* = 0.22; *p* = 0.48	[42]
	Week 3 Weekly load vs URTI symptoms	*r* = 0.18; *p* = 0.57	[42]
	Week 4 Weekly load vs URTI symptoms	*r =* 0.41; *p* = 0.18	[42]
	Week 5 Weekly load vs URTI symptoms	*r =* 0.41; *p* = 1.18	[42]
	Week 6 Weekly load vs URTI symptoms	*r =* 0.02; *p* = 0.94	[42]
	Week 7 Weekly load vs URTI symptoms	*r =* 0.07; *p* = 0.81	[42]
	Week 8 Weekly load vs URTI symptoms	*r =* 0.02; *p* = 0.94	[42]
	Week 1 Weekly monotony vs URTI symptoms	*r =* 0.1; *p* = 0.75	[42]
	Week 2 Weekly monotony vs URTI symptoms	*r =* 0.05; *p* = 0.89	[42]
	Week 3 Weekly monotony vs URTI symptoms	*r =* 0.04; *p* = 0.91	[42]
	Week 4 Weekly monotony vs URTI symptoms	*r =* 0.45; *p* = 0.15	[42]
	Week 5 Weekly monotony vs URTI symptoms	*r =* 0.44; *p* = 0.15	[42]
	Week 6 Weekly monotony vs URTI symptoms	*r =* 0.27; *p* = 0.40	[42]
	Week 7 Weekly monotony vs URTI symptoms	*r =* 0.13; *p* = 0.69	[42]
	Week 8 Weekly monotony vs URTI symptoms	*r =* 0.18; *p* = 0.57	[42]
	Week 1 Weekly strain vs URTI symptoms	*r =* 0.00; *p* = 0.99	[42]
	Week 2 Weekly strain vs URTI symptoms	*r =* 0.07; *p* = 0.81	[42]
	Week 3 Weekly strain vs URTI symptoms	*r =* 0.04; *p* = 0.89	[42]
	Week 4 Weekly strain vs URTI symptoms	*r =* 0.39; *p* = 0.20	[42]
	Week 5 Weekly strain vs URTI symptoms	*r =* 0.49; *p* = 0.10	[42]
	Week 6 Weekly strain vs URTI symptoms	*r =* − 0.17; *p* = 0.59	[42]
	Week 7 Weekly strain vs URTI symptoms	*r =* 0.18; *p* = 0.58	[42]
	Week 8 Weekly strain vs URTI symptoms	*r =* 0.18; *p* = 0.58	[42]
	Week 1 overload training load vs severity of URTI	*p* > 0.05	[53]
	Week 2 overload training load vs URTI	*p* > 0.05	[53]
	Week 1 taper training load vs severity of URTI	*p* > 0.05	[53]
	Week 1 taper training load vs severity of URTI	*p* > 0.05	[53]
	4-week load vs illness	OR = 1.54 [CI 1.13–1.2.12(sic)]; *p* < 0.01*^a^	[90]
	1-week load vs illness	OR = 1.50 [CI 1.13–2.00];* p* < 0.01*	[90]
	4-week ACWR vs illness	OR = 1.10 [CI 0.79–1.52]; *p* = 0.59	[90]
	Prior-day training load vs illness	OR = 1.08 [CI 0.82–1.41]; *p* = 0.57	[90]

*ACWR* acute to chronic work to rest ratio, *OR* odds ratio, *URTI* upper respiratory tract infection

^*^Statistically significant result

^a^Inconsistent or erroneous datum

## Discussion

The aim of this review was to detail the methods of reporting internal and external loads in adolescent athletes and use best-evidence synthesis to report their relationship with changes in physical qualities, injury, or illness. Common internal methods of monitoring load included sRPE, dRPE, HR, and novel scales of perceived intensity, while common external methods of monitoring load included GNSS, resistance training volume, training duration, throw count, and accelerometry. Findings showed there was moderate evidence of a relationship between resistance training volume load and strength, and between training duration and throw count and injury. However, all other relationships between training load and physical qualities, injury, or illness were limited or inconsistent. An indirect finding of this review was the common use of univariate statistical techniques to establish the load–response relationship in adolescent athletes. Whilst the findings of this review indicate limited evidence for most relationships between training load and changes in physical qualities, injury and/or illness, this may be due to highly complex interactions, as opposed to relationships not existing. For example, a number of factors outside of training load, such as sleep, stress, and maturation, will influence these relationships, but were not quantified. Based on the findings and interpretation of this review, it is recommended that researchers and practitioners should consider (1) accounting for resistance training volume load when monitoring strength training; (2) monitoring training duration, and throws, if appropriate, for potential increases in injury risk; (3) assessing factors, such as maturation, that may influence how adolescent athletes respond to load; and (4) the appropriateness of the statistical methodology used to establish a load–response relationship.

### Methods of Monitoring Training Loads

A variety of internal and external load monitoring tools were used, with the distribution between the use of internal (*n* = 32) and external (*n* = 35) methods of monitoring load close to even. The most commonly reported internal load monitoring tools were sRPE and heart rate, whilst the most commonly reported external tools were training duration and GNSS. The prevalence of these methods throughout the literature likely reflects the accessibility and relative ease with which they can be used. For example, sRPE gives an overview of the load of an entire training session and is commonly used to accumulate the load across multiple forms of training (e.g., field-based training and resistance training) [96]. Alternatively, heart rate and GNSS are becoming increasingly accessible for practitioners and help provide greater information regarding the distribution of intensity across a training session [97]. It should be acknowledged, though, that the use of heart rate and GNSS is associated with added expense due to the equipment involved, which may limit its accessibility in adolescent sport. Furthermore, it does require additional expertise to collect and analyse the data appropriately [12]. Additionally, practitioners in adolescent settings are often constrained by both time and financial resources. Therefore, the methods of monitoring training load that are used throughout the adolescent literature may be an outcome of accessibility and relative ease of use rather than their relationship with changes in physical qualities, injury, or illness. Consequently, practitioners and researchers should carefully consider what the monitoring methods that are being used will add to a training environment and also whether the budget and expertise are available to help interpret the subsequent information.

### Training Loads and Physical Qualities

There was moderate evidence of a relationship between resistance training volume and strength, with three studies and 53% of the results indicating a positive relationship and no results indicating a negative relationship. Resistance training volume is a commonly used monitoring tool for strength training and represents the product of the number of repetitions performed multiplied by the external load lifted [98]. Developing strength is recommended throughout all stages of adolescent development [22], as strength can be protective against injuries [5], facilitate performance [4], and underpins the development of other physical qualities, such as power [99]. Despite its importance, limited research (*n* = 4) has reported the relationship between training loads and strength. Additionally, all the studies were observational, limiting the ability for causal inference to be drawn. One of the studies found that a medium-volume group had greater improvements in their snatch 1RM as compared with a low-volume group, but not compared with the high-volume group [55]. These results indicate that there may be an upper limit to the load–response relationship, but this has not been explored in detail in adolescent athletes. Nonetheless, volume load appears to demonstrate the strongest evidence for a relationship with changes in strength in adolescent athletes, and therefore warrants consideration by practitioners.

Increases in strength occur as a result of a combination of neural and muscular factors [100]. In pre-peak height velocity (PHV) athletes, most strength-based adaptations occur as a consequence of increased coordination [22, 101]. Strength increases seen from resistance training volume may be due to greater opportunities to practice. Post-PHV alterations in sex hormones enhance capacity for muscular adaptations, such as hypertrophy, to resistance training [22, 100]. Therefore, although the mechanisms are likely to differ, resistance training volume load should be a focus throughout all stages of adolescent athletic development. This may have practical implications in the programming and periodisation of resistance training in adolescent athletes. However, there is no evidence on how much resistance training volume should be prescribed, and future research should investigate the minimal effective doses.

There were no consistent relationships between training monitoring tools and aerobic fitness across 11 studies. The most commonly reported monitoring tools were sRPE (*n* = 8), GNSS devices (*n* = 5), and heart rate monitors (*n* = 6). Interestingly, a relationship between upper-body resistance training load and 800-m time was found in one study [93], but this relationship is likely to be spurious. Measures of gross volume or load, such as total distance and TRIMPs, may not accurately represent the work performed, as they provide no information as to the distribution of volume or intensity. Some studies provided more informative measures of training load, such as distance and time between speed thresholds, but this did not improve any relationship [47, 51, 86]. The lack of consistent findings may be due to factors that mediate the response to aerobic training, such as maturation [102, 103], changes in body mass [104], and variety in the monitoring tools and testing methods used to assess aerobic fitness [19]. Previously, it has been shown that adolescent athletes may have altered responses to aerobic training throughout maturation [19]. However, no studies investigating the relationship between training loads and aerobic fitness reported the maturation level of the participants. Additionally, numerous training methods can enhance aerobic capacity, such as cross-training modalities (e.g., cycle or rowing ergometers), which may influence the effectiveness of some monitoring tools in accurately assessing overall training load (e.g., GNSS devices). Therefore, practitioners should consider external factors (e.g., maturation and body mass) that may influence aerobic capacity, and all forms of training that are being completed by the athlete.

The ‘Goldilocks’ effect of the load–response relationship was evident in this review, with several studies finding that greater training loads were related to the decreased expression of physical qualities [46, 70, 71, 76, 94]. Given that the athletes in all studies were training throughout the period of investigation, it is unlikely de-training occurred. An alternative explanation for the decreased expression of physical qualities may be that excessive training loads and inadequate recovery caused substantial fatigue within the tested athletes [105], with studies reporting daily training loads as high as 1400 AU, equivalent to > 4.5 h of ‘hard’ training (i.e., > 8 RPE on a CR10 scale) [70, 96]. Interestingly, two studies that found a negative relationship between sRPE and physical qualities were conducted with tennis players on international tours [70, 71]. Travel can influence performance and recovery through factors such as compromised sleep and nutrition [106, 107]. Therefore, although speculative, altered ability to recover may have played a mediating role in the results observed. Practitioners should also be cautious in interpreting a negative relationship between training load and physical qualities as advocating for a decrease in load, as this may hamper long-term athletic development. To state that more training results in decreased expression of physical capacity without offering solutions for reducing this risk, outside of simply reducing load, is unproductive. Instead, an increased focus should be placed on increasing or maintaining training loads whilst protecting athletes from injuries and fatigue by manipulating or accounting for factors that may mediate the load–response relationship.

### Training Loads and Injury

There was moderate evidence of a relationship between training duration and throw count, and injury. However, there are limited applications of this finding as the relationship is likely due to increased exposure to risk. There were no other clear relationships between either internal or external monitoring tools and injury. Different metrics to assess distribution of training load were used, including the ACWR [33, 35, 38, 45, 63, 72, 83, 90], monotony [63, 75], and strain [63, 75]. Analysis of included studies was also affected by inconsistent definitions of injury. For example, methods of reporting injury included reporting of a physical complaint or medical attention [33, 40, 69], time-loss injuries or illness [45, 63, 72, 75, 83, 90], and time loss > 3 weeks [58]. Therefore, the inconsistent collection and analysis of methods used across different studies may unintentionally impede practitioners and researchers from drawing consensus across investigations into training load and injury.

The ACWR was used across seven of the 12 studies that investigated the relationship between internal load measures, such as sRPE, and injury risk [33, 38, 45, 63, 72, 83, 90]. The ACWR is a monitoring method that quantifies the acute changes in training load (e.g., most recent 7 days) relative to chronic training load (e.g., most recent 28 days) [108]. However, there are inconsistent approaches to calculating the ACWR, including variable time frames and different statistical approaches, such as exponentially weighted moving averages or rolling averages [109], and coupled or uncoupled chronic workloads [110]. The different statistical methods used to calculate ACWR can substantially alter the outcome, with one study demonstrating that quadratic calculation of the relationship between ACWR and injury was statistically significant, whereas linear was not [63]. Additionally, methodological pitfalls associated with the ACWR have been highlighted in studies that show that actual training loads confer no greater predictive value for injury risk than random chronic training loads [111]. Therefore, there is limited evidence for the use of ACWR as a metric to guide decisions around injury risk in an adolescent load monitoring programme.

The monitoring tool with the strongest relationship between training load and injury was training duration, with 15 of 17 studies investigating this and 56% of contributing findings indicating a positive relationship. However, the use of various methods of reporting training duration makes it difficult to draw conclusions. For example, some studies examined training duration in the previous week [57], fortnight [39], over a season [66], weekly change in training duration [68], or duration relative to age [79, 82]. Whilst there were inconsistencies in the reporting mechanism, there remains moderate evidence that increased training duration in preceding periods increases injury risk. Superficially, this finding may have practical applications as training duration is simple to collect and easy to analyse [12]. However, this relationship is likely due to athletes having greater risk of injury simply due to increased exposure. It should be noted that despite the potential for a greater number of injuries, training is necessary to develop physical qualities, tactical knowledge, and technical skills. Finding a balance between training exposure and athletic development is needed. Whilst this may be the focus of future research, it may be difficult to generalise research-based results to specific populations, due to the multi-factorial nature of injury.

Overall, there was limited evidence of a relationship between training loads and injury risk in adolescent athletes. Furthermore, training load, when administered appropriately, may also be protective against injury, highlighting the ‘Goldilocks’ effect [11]. Therefore, practitioners should exercise caution when using singular training loads to assess injury risk in adolescent athletes in isolation from mediating factors. Other factors that should be considered when assessing injury risk may include sleep, stress, nutrition, biomechanics, and injury history [112]. However, this list is non-exhaustive, and the highly complex nature of injuries means that identifying and accounting for all risk factors in an applied setting is difficult.

### Training Loads and Illness

The evidence of a relationship between training loads and illness was limited or inconsistent with only six studies investigating these outcomes and only 4.6% of contributing findings indicating a relationship between training load and illness. The body interprets exercise as a stressor, similar to other psychological and physiological stressors [113]. Short-term periods of stress are thought to be immunoprotective, whereas prolonged exposure to stress is immunosuppressive [113]. Interestingly, the two studies that found a significant relationship between training load and illness had the longest observational period of any included studies (20 weeks and two seasons) [40, 90]. Given the delayed relationship between prolonged periods of high stress and illness, studies of insufficient length may have confounded the results of the best-evidence synthesis. However, it is not known what amount of exposure to excessive stress increases the risk of illness. Additionally, given the general nature of stress, other stressors that adolescent athletes face, such as academic, social, and performance pressure, will likely contribute to this relationship, and should be accounted for [114].

## Limitations and Future Directions

The results of this review provide important considerations for researchers and practitioners investigating and monitoring the training loads of adolescent athletes. However, there are limitations within this review that should be considered before implementing the findings. A limitation of the best-evidence synthesis methodology was the use of ‘vote-counting’ criteria, with no weightings applied to the magnitude of the stimulus or strength of the relationship [32]. Vote counting was used due to the lack of a validated method of quantifying stimulus magnitude and strength of relationships across different load monitoring tools and heterogeneous statistical methodologies. While standardisation of reporting training load metrics may assist in facilitating future meta-analyses, it is unlikely that a consistent framework will be universally adopted, due to barriers such as variation in the appropriateness of different metrics between sports, advances in technology, practitioner preferences, and the ever-increasing number of methods used to quantify training load. Additionally, a key consideration for training adolescent athletes is the effect of maturation on the response to training [19]. However, only four studies reported the maturation levels of their participants, limiting the ability to draw conclusions on the response to training load at different stages of adolescence. Previously, it has been shown that using chronological age as a surrogate for maturation is flawed as adolescents mature at different rates [115]. Given that maturity status can be assessed with relative ease (e.g., peak height velocity [116]), researchers may wish to consider reporting these data when investigating adolescent populations. This information would help inform future research on the role of maturation in the load–response relationship.

The lack of consistent findings in this review may be due to the multi-factorial nature of the load–response relationship. The individual response to training load is both positively and negatively influenced by factors such as physical qualities [6, 117, 118], stress [119], sleep [120], nutrition [121], and academic stress [122]. For example, one study found that self-esteem, sleep, and nutrition altered the injury rates in adolescent athletes in a multi-sport cohort [89]. It has also been demonstrated that increased stress levels correlate to a reduced adaptation to aerobic training [119]. The heterogeneity of the included studies and the complex nature of any latent relationship may have caused further noise in attempting to establish relationships with training load. The ability to adequately recover from a training dose is inextricably linked to non–training-related factors. Therefore, the ‘Goldilocks’ effect should not be viewed as solely being related to load. However, it is not feasible to accurately measure all of the factors that may influence the response to training load. Instead, practitioners may be best served to understand that rapid increases in stress, or prolonged periods of excessive stress, are likely to have negative outcomes and proactively modify loads accordingly.

To address the complex nature of the load–response relationship, it has recently been proposed that advanced statistical methods may be appropriate [123]. Most studies included in this review used logistic and linear regression methodologies, which are bound by fairly stringent assumptions (e.g., normality of residuals, homogeneity of variance) and are susceptible to issues such as multicollinearity [124]. These limitations may be accounted for by using alternative statistical techniques such as dimension reduction or feature selection algorithms. Compared with univariate correlation analysis, statistical methodologies that use dimension reduction (such as principle component analysis) or feature selection algorithms (such as elastic net regressions) may be more appropriate to establish a load–response relationship. By accounting for multi-collinearity, these techniques may be less likely to report spurious correlations. These techniques have previously been used to establish the relationship between training load and changes in aerobic fitness in adult athletes [124], as well as for talent identification [125]. Consequently, it is recommended that researchers consider the appropriateness of the statistical technique used when attempting to establish a dose–response relationship.

## Conclusion

This systematic review is the first to investigate and detail the relationships between internal and external methods of monitoring training load and their relationship with changes in physical qualities, injury, or illness in adolescent athletes. The most commonly reported monitoring tools were sRPE and training duration. There was moderate evidence of a relationship between resistance training volume load and strength, and between throw count, training duration, and injury. However, all other relationships were either limited or inconsistent. The lack of consistent or strong relationships with load monitoring tools is likely due to the complex, individualised response to training load. Furthermore, whilst there was a general trend that greater training duration increased injury risk, inconsistencies in the reporting of training duration, and injury definitions, makes drawing conclusions difficult, and there is limited practical application of this finding. This systematic review’s lack of clear trends is potentially due to the univariate nature of the data provided, which fails to account for the complex nature of any relationship between load and training outcomes where numerous mediating factors likely influence the load–response relationship. Therefore, researchers may wish to assess the interactions between multiple training loads through advanced statistical methods and their outcomes and consider mediating factors, such as maturation, that may influence this relationship.

Based on the current evidence, resistance training volume appears to be the best load monitoring tool for improving strength in adolescent athletes. Collecting resistance training volume is highly practical, requires relatively few resources to collect, and is simple to analyse. Throw count and training duration may also be valuable to assess injury risk in sports where they are applicable. Whilst the development of strength should be a key focus of adolescent development [22], this measure is only relevant to resistance training and likely only captures a small portion of the adolescent monitoring puzzle. As such, other methods are needed to quantify training and non-training stressors that are likely to influence training outcomes.

## Supplementary Information

Below is the link to the electronic supplementary material.

Supplementary file1 (DOCX 32 kb)

## Declarations

### Availability of data and material

All data and material reported in this systematic review are from peer-reviewed publications.
