# Supplementary material for: Methods of Monitoring Internal and External Loads and Their Relationships with Physical Qualities, Injury, or Illness in Adolescent Athletes: A Systematic Review and Best-Evidence Synthesis
Source: Sports Med. 2023 Apr 18;53(8):1559–93. doi: 10.1007/s40279-023-01844-x (PMC10356657; doi:10.1007/s40279-023-01844-x)
Supplement: Supplementary file 1 — Supplementary file1 (DOCX 32 kb) [file 40279_2023_1844_MOESM1_ESM.docx]

| **Supplementary Material 1.** Questions selected from the Modified Downs and Black checklist | |
| --- | --- |
| Question Number | Question |
|  | **Reporting** |
| 1 | Is the hypothesis/aim/objective of the study clearly described? |
| 2 | Are the main outcomes to be measured clearly described in the introduction or methods section? |
| 3 | Are the characteristics of the subjects included in the study clearly described? |
| 4 | Are the main findings of the study clearly described? |
| 5 | Does the study provide estimates of the random variability in the data for the main outcomes? |
| 6 | Have actual probability values been reported (e.g., 0.035 rather than < 0.05) for the main outcomes except where the probability value is < 0.001? |
|  | **External validity** |
| 7 | Were the subjects asked to participate in the study representative of the entire population from which they were recruited? |
| 8 | Were those subjects who were prepared to participate representative of the entire population from which they were recruited? |
|  | **Internal validity bias** |
| 9 | If any of the results of the study were based on “data dredging,” was this made clear? |
| 10 | Were the statistical tests used to assess the main outcomes appropriate? |
| 11 | Were the main outcome measures accurate (valid and reliable)? |
|  | **Power** |
| 12 | Did the study have sufficient power to detect a clinically important effect  where the probability value for a difference being due to chance is less than  5%? |

| **Supplementary material 2.** Results of modified Downs and Black for included studies | | | | | | | | | | | | | | | |
| --- | --- | --- | --- | --- | --- | --- | --- | --- | --- | --- | --- | --- | --- | --- | --- |
| **References** | **Reporting** | | | | **External Validity** | | | | **Internal validity bias** | | | **Power** | | | **Total** |
|  | **1** | **2** | **3** | **4** | | **5** | **6** | **7** | | **8** | **9** | **10** | **11** | **12** |  |
| Ahmun et al., [33] | 1 | 1 | 1 | 1 | | 1 | 1 | 0 | | 1 | 1 | 1 | 1 | 0 | 10 |
| Akubat et al., [34] | 1 | 1 | 0 | 1 | | 1 | 0 | 0 | | 0 | 1 | 1 | 1 | 0 | 7 |
| Albrecht et al., [35] | 0 | 1 | 1 | 1 | | 1 | 1 | 0 | | 0 | 1 | 1 | 1 | 0 | 8 |
| Antualpa et al., [36] | 1 | 1 | 1 | 1 | | 1 | 1 | 0 | | 0 | 1 | 1 | 1 | 0 | 9 |
| Bacon and Mauger [37] | 1 | 1 | 1 | 1 | | 1 | 1 | 0 | | 0 | 1 | 1 | 1 | 0 | 9 |
| Bowen et al., [38] | 0 | 1 | 1 | 1 | | 0 | 0 | 0 | | 0 | 1 | 1 | 1 | 0 | 6 |
| Brink et al., [39] | 0 | 1 | 1 | 1 | | 1 | 0 | 0 | | 0 | 1 | 1 | 1 | 0 | 7 |
| Brink et al., [40] | 1 | 0 | 0 | 1 | | 1 | 0 | 0 | | 0 | 1 | 1 | 1 | 0 | 6 |
| Brisola et al., [41] | 1 | 1 | 1 | 1 | | 1 | 0 | 0 | | 0 | 1 | 1 | 1 | 1 | 9 |
| Brunelli et al., [42] | 0 | 1 | 1 | 1 | | 1 | 1 | 0 | | 0 | 1 | 1 | 1 | 0 | 8 |
| Cahalan et al., [43] | 0 | 1 | 0 | 1 | | 1 | 1 | 0 | | 0 | 1 | 1 | 1 | 0 | 7 |
| Chaabene and Negra, [44] | 0 | 1 | 1 | 1 | | 1 | 1 | 0 | | 0 | 1 | 1 | 1 | 0 | 8 |
| Delecroix et al., [45] | 0 | 1 | 1 | 1 | | 1 | 1 | 0 | | 0 | 1 | 1 | 1 | 0 | 8 |
| Dobbin et al., [46] | 0 | 1 | 1 | 1 | | 1 | 1 | 0 | | 0 | 1 | 1 | 1 | 0 | 8 |
| Ellis et al., [47] | 0 | 1 | 1 | 1 | | 1 | 0 | 0 | | 0 | 1 | 1 | 1 | 0 | 7 |
| Fett et al., [48] | 0 | 1 | 1 | 1 | | 1 | 0 | 0 | | 0 | 1 | 1 | 0 | 0 | 6 |
| Figueiredo et al., [49] | 1 | 1 | 1 | 1 | | 1 | 1 | 1 | | 1 | 1 | 1 | 1 | 0 | 11 |
| Figueiredo et al., [50] | 1 | 1 | 1 | 1 | | 1 | 1 | 0 | | 0 | 1 | 1 | 1 | 1 | 10 |
| Fitzpatrick et al., [51] | 1 | 1 | 1 | 1 | | 1 | 1 | 0 | | 0 | 1 | 1 | 1 | 0 | 9 |
| Fleisig at al., [52] | 1 | 1 | 0 | 1 | | 1 | 1 | 1 | | 1 | 1 | 1 | 1 | 0 | 10 |
| Freitas et al., [53] | 1 | 1 | 1 | 1 | | 1 | 0 | 0 | | 0 | 1 | 1 | 1 | 0 | 8 |
| Gil-Rey et al., [54] | 1 | 1 | 1 | 1 | | 1 | 1 | 0 | | 1 | 1 | 1 | 1 | 0 | 10 |
| Gonzalez-Badillo et al., [55] | 1 | 1 | 1 | 1 | | 0 | 1 | 0 | | 0 | 1 | 1 | 1 | 0 | 8 |
| González-Badillo et al., [56] | 1 | 1 | 1 | 1 | | 0 | 1 | 0 | | 0 | 1 | 1 | 1 | 0 | 8 |
| Hartwig et al., [57] | 0 | 1 | 1 | 1 | | 1 | 1 | 0 | | 0 | 1 | 1 | 1 | 0 | 8 |
| Huxley et al., [58] | 0 | 1 | 0 | 1 | | 1 | 1 | 1 | | 0 | 1 | 1 | 0 | 0 | 7 |
| Johannsson et al., [59] | 1 | 1 | 1 | 1 | | 1 | 1 | 1 | | 1 | 1 | 1 | 1 | 0 | 11 |
| Johansson et al., [60] | 1 | 1 | 1 | 1 | | 1 | 1 | 1 | | 1 | 1 | 1 | 1 | 0 | 11 |
| Jones et al., [61] | 0 | 1 | 1 | 0 | | 1 | 1 | 1 | | 1 | 0 | 0 | 0 | 0 | 6 |
| Kiernan et al., [62] | 0 | 1 | 1 | 0 | | 0 | 1 | 0 | | 0 | 1 | 1 | 1 | 0 | 6 |
| Lathlean et al., [63] | 1 | 1 | 1 | 1 | | 1 | 1 | 1 | | 1 | 1 | 1 | 1 | 0 | 11 |
| Lopez Segovia et al., [64] | 0 | 1 | 1 | 1 | | 1 | 0 | 0 | | 0 | 1 | 1 | 1 | 0 | 7 |
| Lyman et al., [65] | 0 | 1 | 0 | 1 | | 0 | 1 | 1 | | 1 | 1 | 1 | 1 | 1 | 9 |
| Martínez-Silván et al., [66] | 0 | 1 | 1 | 1 | | 0 | 0 | 0 | | 0 | 1 | 1 | 1 | 0 | 6 |
| Mehta et al., [67] | 1 | 1 | 1 | 1 | | 1 | 0 | 1 | | 1 | 1 | 1 | 1 | 1 | 11 |
| Møller et al., [68] | 1 | 1 | 1 | 1 | | 1 | 1 | 1 | | 0 | 1 | 1 | 1 | 0 | 10 |
| Moreno-Pérez et al., [69] | 0 | 1 | 1 | 1 | | 1 | 1 | 0 | | 0 | 1 | 1 | 1 | 1 | 9 |
| Murphy et al., [70] | 1 | 1 | 1 | 1 | | 1 | 0 | 0 | | 0 | 1 | 1 | 1 | 0 | 8 |
| Murphy et al., [71] | 1 | 1 | 1 | 1 | | 1 | 0 | 0 | | 0 | 1 | 1 | 1 | 0 | 8 |
| Myers et al., [72] | 1 | 1 | 1 | 1 | | 1 | 1 | 0 | | 0 | 1 | 1 | 1 | 0 | 9 |
| Nobari et al., [73] | 0 | 1 | 0 | 1 | | 1 | 1 | 0 | | 0 | 1 | 1 | 1 | 0 | 7 |
| Nobari et al., [74] | 0 | 1 | 1 | 1 | | 1 | 1 | 0 | | 0 | 1 | 1 | 1 | 1 | 9 |
| O'Keeffe et al., [75] | 0 | 1 | 1 | 1 | | 1 | 1 | 0 | | 0 | 1 | 1 | 1 | 0 | 8 |
| Otaegi and Arcos, [76] | 0 | 1 | 1 | 1 | | 1 | 1 | 0 | | 0 | 1 | 1 | 1 | 0 | 8 |
| Patel et al., [77] | 1 | 1 | 1 | 1 | | 1 | 1 | 1 | | 0 | 1 | 1 | 1 | 0 | 10 |
| Post et al., [78] | 1 | 1 | 1 | 1 | | 1 | 1 | 0 | | 0 | 1 | 1 | 1 | 0 | 9 |
| Post et al., [79] | 1 | 1 | 1 | 1 | | 1 | 1 | 0 | | 0 | 1 | 1 | 1 | 0 | 9 |
| Prieto-González et al., [80] | 1 | 1 | 0 | 1 | | 1 | 1 | 1 | | 1 | 1 | 1 | 1 | 0 | 10 |
| Pullinger et al., [81] | 1 | 1 | 1 | 1 | | 0 | 1 | 0 | | 0 | 1 | 1 | 1 | 0 | 8 |
| Purnell et al., [82] | 0 | 1 | 1 | 1 | | 0 | 1 | 0 | | 0 | 1 | 1 | 1 | 1 | 8 |
| Raya-González et al., [83] | 1 | 1 | 1 | 1 | | 1 | 0 | 0 | | 0 | 1 | 1 | 1 | 1 | 9 |
| Sawczuk et al., [84] | 0 | 1 | 1 | 1 | | 1 | 0 | 0 | | 0 | 1 | 1 | 1 | 0 | 7 |
| Sugimoto et al., [85] | 1 | 1 | 1 | 1 | | 1 | 1 | 0 | | 0 | 1 | 1 | 1 | 0 | 9 |
| Taylor et al., [86] | 0 | 1 | 1 | 1 | | 1 | 1 | 0 | | 0 | 1 | 1 | 0 | 0 | 7 |
| Visnes and Bahr, [87] | 0 | 1 | 1 | 1 | | 1 | 1 | 0 | | 1 | 1 | 1 | 1 | 0 | 9 |
| Von Rosen et al., [88] | 1 | 1 | 1 | 1 | | 1 | 0 | 0 | | 1 | 1 | 1 | 1 | 0 | 9 |
| von Rosen et al., [89] | 1 | 1 | 1 | 1 | | 1 | 1 | 0 | | 1 | 1 | 1 | 1 | 0 | 10 |
| Watson et al., [90] | 0 | 1 | 1 | 1 | | 1 | 1 | 0 | | 1 | 1 | 1 | 1 | 0 | 9 |
| Weakley et al., [91] | 1 | 1 | 1 | 1 | | 1 | 1 | 0 | | 0 | 1 | 1 | 1 | 0 | 9 |
